# Population Distribution Analyses Reveal a Hierarchy of Molecular Players Underlying Parallel Endocytic Pathways

**DOI:** 10.1371/journal.pone.0100554

**Published:** 2014-06-27

**Authors:** Gagan D. Gupta, Gautam Dey, Swetha MG, Balaji Ramalingam, Khader Shameer, Joseph Jose Thottacherry, Joseph Mathew Kalappurakkal, Mark T. Howes, Ruma Chandran, Anupam Das, Sindhu Menon, Robert G. Parton, R. Sowdhamini, Mukund Thattai, Satyajit Mayor

**Affiliations:** 1 National Centre for Biological Sciences, Tata Institute of Fundamental Research, UAS/GKVK Campus, Bangalore, India; 2 The University of Queensland, Institute for Molecular Bioscience, Queensland, Australia; Iowa State University, United States of America

## Abstract

Single-cell-resolved measurements reveal heterogeneous distributions of clathrin-dependent (CD) and -independent (CLIC/GEEC: CG) endocytic activity in *Drosophila* cell populations. dsRNA-mediated knockdown of core versus peripheral endocytic machinery induces strong changes in the mean, or subtle changes in the shapes of these distributions, respectively. By quantifying these subtle shape changes for 27 single-cell features which report on endocytic activity and cell morphology, we organize 1072 *Drosophila* genes into a tree-like hierarchy. We find that tree nodes contain gene sets enriched in functional classes and protein complexes, providing a portrait of core and peripheral control of CD and CG endocytosis. For 470 genes we obtain additional features from separate assays and classify them into early- or late-acting genes of the endocytic pathways. Detailed analyses of specific genes at intermediate levels of the tree suggest that Vacuolar ATPase and lysosomal genes involved in vacuolar biogenesis play an evolutionarily conserved role in CG endocytosis.

## Introduction

Endocytosis occurs by multiple means at the cell surface [1], [2]. In addition to the clathrin-dependent (CD) endocytic pathway, numerous clathrin-independent endocytic mechanisms continue to be discovered [3]. These include a pinocytic pathway called the CLIC/GEEC (CG) pathway [4], which is responsible for internalizing a large fraction of the fluid phase, many GPI-anchored proteins, and some specific cell surface proteins such as CD44 and G-protein coupled receptors [4]–[6]. The CG system is a high capacity pathway evolutionarily conserved throughout metazoa [7], [8], implicated in plasma membrane homeostasis and the regulation of signaling [4], [5]. Many viruses and toxins have been shown to use the CG pathway for their productive entry into cells [3]. Ultrastructural analyses show that this endocytic process is initiated by a pleomorphic set of clathrin-independent endocytic carriers (CLICs; [9]) that eventually fuse with distinct early endosomal compartments called GEECs (GPI-anchored protein enriched endosomes) [10], [11]. Membrane and volume components of these endosomal compartments are recycled to the cell surface [12], [13], or addressed to a variety of cargo-specific locations [4] including the sorting endosome, which is the major recipient of cargo from the CD endocytic pathway [11].

Recent experiments suggest that the CG pathway involves a specific type of actin polymerization controlled via the regulated cycling of a Rho family GTPase, Cdc42 [10], [12]. Endocytosis is initiated by the recruitment of Arf1 and an Arf1-binding RhoGAP protein called ARHGAP10 [14]. The activity of Arf1 itself appears to be regulated by a GEF called GBF1 at the cell surface [8]. The Bar-domain protein GRAF1 participates in the post-endocytic dynamics of the GEECs [15]. It is clear that a host of other core and peripheral molecules must drive the trafficking of these compartments and their cargo to specific destinations inside the cell. Here we exploit a powerful analytic approach, based on the phenomenon of cellular heterogeneity, to detect the strong contributions of core machinery as well as the subtle contributions of peripheral components. Using this approach, we build a comprehensive molecular portrait of CD and CG endocytic pathways acting in concert in single cells.

We recently demonstrated the ease of RNAi-based gene perturbation in *Drosophila* S2R+ cells to identify new components of the CG pathway [8]. We have adapted this assay to an array format employing millimeter-scale wells printed on borosilicate slides, allowing us to image hundreds of cells per well and screen hundreds of genes per slide. Cell arrays increase throughput at the cost of introducing systematic measurement artifacts of unknown origin, in the form of edge effects and column or row biases [16], [17]. Standard analyses adopt a conservative approach in which only strong perturbations are selected as hits – for example RNAi knockdowns which produce factors of two or greater changes in the population-averaged value of some feature of interest. We eliminate this problem by studying the entire population distribution of measured features. Broad population distributions are hallmarks of cellular heterogeneity, manifest in any single-cell-resolved measurement. Studies in bacteria, yeast, and metazoan cell lines have shown this heterogeneity to arise partly through intrinsic stochastic mechanisms – for example, population-wide variations in endocytic rates have been traced to stochastic fluctuations in gene expression, and to inter-cell interactions [18]–[20]. Population distributions therefore contain information about the underlying biology, and our assay is designed to exploit this fact: we measure single-cell-resolved population distributions of a range of features related to endocytic activity, and quantify changes to these distributions under RNAi knockdowns to identify strong as well as subtle hits.

We have examined over half the *Drosophila* genome – 7131 genes that share significant homology with their mammalian counterparts – and quantified their contributions to various aspects of CD and CG endocytosis. We report a large number of hits (1072 genes), over half of which are statistically demonstrated to be true positives. These hits survive validation in independent assays, and their effects in S2R+ cells are recapitulated in *Drosophila* mutants as well as mammalian cell lines. A tree-based analysis of all the hits provides a rich portrait of core and peripheral control of parallel endocytic processes, while follow-up studies on a subset of candidate genes reveal new molecular details on their mode of action.

## Results

### Tracking endocytic activity in single cells

**Figure 1:**
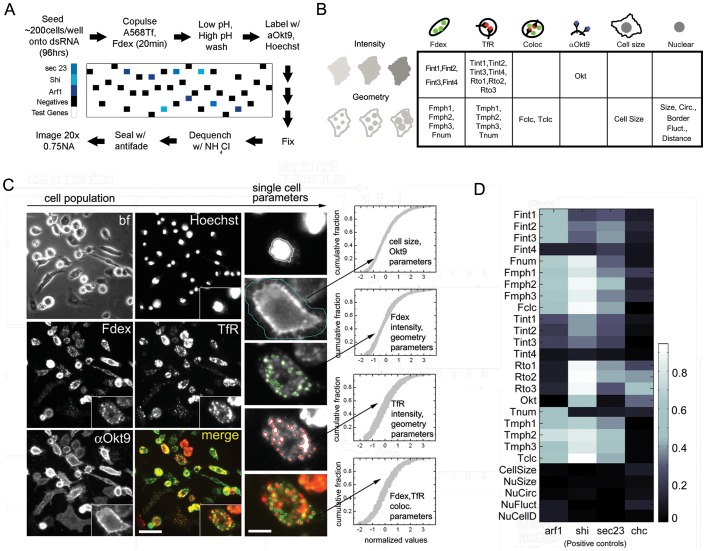
Quantitative profiling of two endocytic routes at single cell resolution. (A) Experimental workflow outline for cell seeding, transfection and multiplex endocytic assays to obtain multifeature data across 7131 gene depletions. The entire procedure was performed on a cell array (see Figure S1A; details in SOM) and the positions of negative and positive (dsRNA against *sec23*, *arf1*, *shi*) controls are highlighted in their respective colours. (B) Table grouping the 27 quantitative features into categories. The top half of the table contains direct measurements of intensity, while the lower half contains geometric parameters of the cell, endosomes and nucleus. Various measurements are made from each fluorescent channel, including those utilizing different pixel radii for local background subtraction while detecting endosomes. (C) Representative brightfield (bf) and fluorescent micrographs of a field of view of individual cells (zoomed in insets) labeled with four different fluorescent probes: Hoechst; FITC-Dextran (Fdex); Alexa568-Tf (Tf); Alexa647-αOkt9 (Okt9); (see Methods S1 for details). The psuedocolour merge image is a composite of the Fdex (green), TfR (red) and Okt9 (blue) channels. Scale bar = 15 µm; inset = 3×. (D) Grayscale heatmap representing the fraction of four control genes (*arf1* (*arf79f*); *shi*; *sec23*; *chc*) picked up as hits (above a Z-score threshold of 3) across all 27 features in the entire dataset. Higher values on the grayscale bar denote higher pick-up rates. The features with higher pick-up rates correspond to the known endocytic roles of these genes.

Our cell array platform (Figure **1A**; Figure **S1A**) provides single-cell-resolved population distributions of multiple endocytic features under thousands of dsRNA-mediated gene knockdowns. RNAi was performed in triplicate on a 10×30 array of millimeter-scale wells printed on borosilicate slides: 30 wells were negative controls; 8 were positive controls; and the remainder contained dsRNA targeting individual genes. We targeted a set of 7131 *Drosophila* genes enriched in phylogenetically conserved ‘metagenes’ (>80% coverage; [21]; Figure **S1E**). A few genes were tested multiple times as internal technical controls (Table **S1**); throughout our analysis these are treated as if they were distinct genes, giving a total of 7216 knockdowns tested in triplicate.

The CD and CG pathways are simultaneously active in *Drosophila* S2R+ cells [7], [8], which detectably express most of our candidate genes (Figure **S1F**). We used a pulse-labeling protocol, fluorescence microscopy and image-analysis to follow the simultaneous activity of parallel endocytic pathways in this cell line. We pulsed fluorophore-labeled dextran (Fdex) and Transferrin (Tf) to track the CG pathway and the Transferrin receptor (TfR)-mediated CD pathway, respectively (Figure **1A**).Our high-resolution images allowed us to extract multiple quantitative features for each cell (Collinet et al., 2010). We computed 27 features as follows: five quantified cell and nuclear geometry; eight quantified aspects of endosomal intensity (F/T int1– int4), morphology or geometry (F/T mph1–mph3), and number (F/T num) for each pathway separately; two quantified the co-localization of the two probes (F/T clc); and finally, four were related to externalized TfR levels (Okt9, Rto1 – Rto3) (Figure **1B** and Table **S1**).

### Population distribution shapes are information rich

To screen for hits we require a summary statistic – a compression of the entire population distribution into a single number that can clearly distinguish positive from negative controls. We found that means and variances – popular summary statistics – suffered from systematic slide-positional artifacts, and even differed significantly between negative control wells on the same slide. However, geometric features were typically more robust and less susceptible to artifacts than intensity features. Importantly, we noticed that differences in the shapes of feature distributions between positive and negative control wells were visible even after normalizing out mean and variance, suggesting that distribution shapes alone were sufficient to detect perturbations due to RNAi [22].

### A summary statistic based on distribution shapes

The idea of extracting information from distribution shapes has a distinguished pedigree ([19]). For example, landmark studies in the early 1940s on photon counting in the human eye [23] and on spontaneous mutations in bacteria [24] relied on the shape of the Poisson distribution. More recently, protein expression distributions have been used to study transcriptional regulation mechanisms in bacteria and yeast [25]; and intrinsic morphological variability has revealed molecular determinants of metazoan cell shape [26], [27]. What distinguishes our approach from all these is that we have no prior model-based expectation of what the distribution shapes should be; we only look for shape changes upon RNAi knockdown.

Skewness and kurtosis report on distribution shapes, but are difficult to estimate for small cell populations. Instead, we calculate a Z-score for each gene based on a modified Kolmogorov-Smirnov (KS) statistic (derived from the maximum vertical deviation between two cumulative distribution functions [28]). This Z-score serves as a quantitative measure of the distribution shape changes between test and negative control wells. Importantly, we only use a subset of negative wells to calculate the Z-score, and use the remaining negative wells to evaluate its performance. Figure S**2A** shows the cumulative distribution functions (cdfs) of all the negative wells for a single feature, Fint 3. We see that positive controls can be easily distinguished from negative controls; and though most target genes resemble negative controls, some show high Z-scores and are likely to represent ‘hits’ [22].

We calculated three Z-scores for every gene (the entire screen was carried out in triplicate) and pooled these data over all genes tested. The number of hits selected from the screen was compared to the number of hits selected from randomly permuted genes as the Z-score threshold was varied. For each threshold value, a gene was considered a hit if two or more of the replicates produced Z-scores above the threshold. The study revealed the presence of reproducible hits in the dataset at a threshold of 3 across all features. Statistical analysis demonstrates that our assay is characterized by a single false positive (FP) rate (a property of the negative controls) but a broad range of true positive (TP) rates (related to the varying degrees of influence different genes can have on the phenotype of interest). Across features, a Z-score threshold of 3 corresponds to FP<0.1 and TP ∼0.5 for single measurements; the FP rate is lower and the TP rate higher if we use triplicate data with a 2/3 rule (Figure S2B; [22]). Thus, our analysis is clearly able to detect weak hits, those with lower TP rates than strong ones, but which might nevertheless be biologically relevant.

Using this strategy we obtain a set of 1072 reliable hits(1072 unique CG identifiers, 1070 unique gene symbols) affecting one or more of the 27 measured features (Table **S1**; also listed at RNAi Data Base http://rnai.ncbs.res.in/endosite). These genes span the entire range of a traditional Z-score derived from measuring the mean and standard deviations of triplicates of the RNAi-treated cell populations compared to their respective negative and positive controls [22]. Meta analysis of the hit set indicates that there is no specific enrichment of putative ‘off targets’ and that metagenes are enriched in this assay (Figure **S1E**), consistent with the perturbation of an evolutionarily conserved pathway.

### Genes affect multiple features simultaneously

Each hit is characterized by a set of 27 Z-scores (in triplicate) which we will refer to as the gene's perturbation vector (PV). Examination of PVs across hits allows us to assess how gene knockdowns can affect multiple aspects of endocytosis simultaneously. For example, the PVs of our positive controls, Arf1 and Sec23 for the CG pathway, and dynamin (encoded by *shibire*) for the CD pathway summarize the perturbation spectrum of these genes (Figure **1D**). The PV for Arf1 (a known regulator of the CG pathway), shows that this gene significantly and specifically affects 7 of the 27 features across a wide range of thresholds: Fdex-marked endosomal intensity (Fint1–3), and endosomal geometry (Fmph1–3); and a Tf geometry feature. This is in perfect agreement with the known roles of Arf1 in regulating the size and number of pinocytic early endosomes, as well as its role in Tf recycling, but not Tf uptake [8], [14]. Similar to Arf1, the depletion of Sec23 (a previously unknown regulator of the CG pathway identified in our laboratory) affects primarily Fdex features, with some influence on Tf geometry features. The effect of Sec23 on the CG pathway is evolutionarily conserved (as has been shown for Arf1 [8]), as demonstrated by assaying the knockdown of human SEC23 in human AGS cells (Figure **S3**). The PV for *Drosophila* dynamin (encoded by *shibire*) deviates primarily in intensity and geometry features for Tf, according to its expected role in regulating the internalization of the Tf receptor. In addition it affects the geometry of endosomal features for the CG pathway, but not the extent of fluid uptake, consistent with our previous studies [7], [8], [10]. Comparably, the *Drosophila* gene encoding Clathrin heavy chain (*chc*) affects largely the quantitative uptake of Tf, which is revealed by measuring the ratio of internalized Tf to the external levels of available TfR for each cell.

### Hits can be organized hierarchically based on the features they perturb

We compressed the triplicate PVs of each gene into a 27-dimensional binary feature vector (FV) as follows: an entry is ‘1’ if the gene is a hit for that parameter (at least two out of three Z-scores is above threshold) and ‘0’ otherwise. Across 7131 dsRNAs there were 1072 hits: 470 influenced fluid-phase uptake, 602 influenced TfR uptake, 267 influenced nuclear morphology, and 26 influenced cell size (Tables **S1**, **S2**).

**Figure 2:**
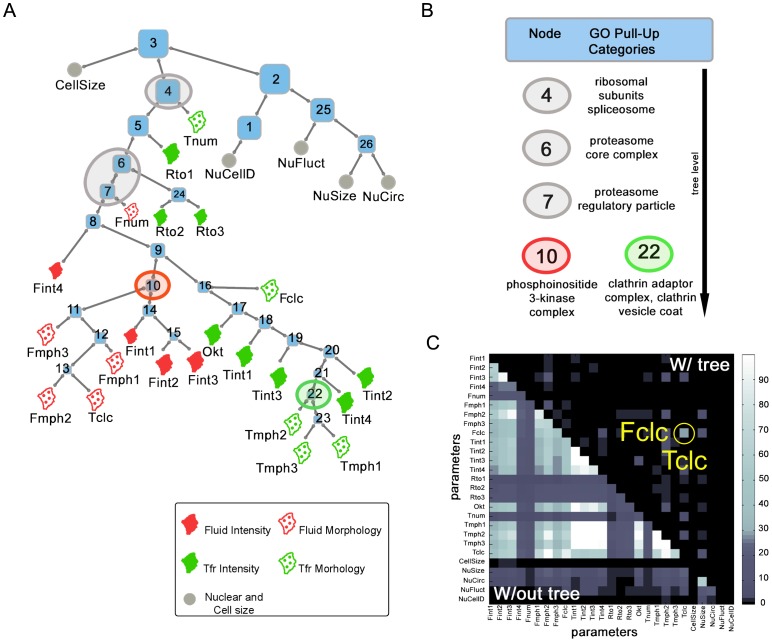
A hierarchical organization of endocytic hits. (A) Maximum parsimony dendogram constructed by binarizing and clustering the perturbation vectors (PV) which show which features each gene is known to perturb. The root node (node 3) is the shared bifurcation point above three main branches – these correspond to genes affecting endocytic pathways, cell size and nuclear morphology PV features. Housekeeping genes such as RNA polymerase and proteasomal subunits populate the stem of the endocytic branch (nodes 4–7) – these affect endocytic processes as a whole. The lower endocytic nodes are further split into CG and CD specific nodes at node 9. (B) Gene Ontology (GO) annotation terms were overlaid onto the tree structure, and GO terms that were present in more than one node were allowed to rise if these nodes were connected. The highest node at which a GO term was found at is shown as a GO ‘Pull-up’ category. For instance, highest node at which the clathrin adaptor complex and clathrin vesicle coat GO terms rise to is node 22, which is the central node specific for CD endocytosis. (C) Grayscale heatmap representing extent of overlaps between all pairs of leaves (colorbar depicts the fraction of overlapping genes) of the leaves prior to (lower diagonal), and post (upper diagonal) tree construction. Most genes which overlapped prior to tree construction have risen to internal nodes. Post the tree construction, significant overlap is seen only between Fclc and Tclc nodes (circled in yellow).

It is expected that the FVs of genes with closely related roles in cellular processes would strongly overlap, and conversely, features that are similar or share genetic control would have more genes in common. Strikingly, we saw that gene knockdowns seemed to affect nested sets of features. Motivated by this, we used a parsimony approach to organize features into a tree (Figure **2A**; Experimental Procedures), formalizing the idea of a hierarchical or nested structure. The 27 leaves of the tree correspond to the 27 measured features. These features are collected into nested groups, each corresponding to an internal node of the tree, all the way to the root. Each node contains a set of genes that affect all the features in the corresponding group; a gene influencing more than one feature is placed higher up the hierarchy. Thus, perturbations in specific features can be examined in the context of the entire dataset. This method of clustering of FVs does not require any prior input or training sets as guides for automated classification, and therefore provides an unbiased description of the dataset.

We have seen that individual genes can affect multiple features simultaneously; the tree allows such genes to migrate upwards, to the internal nodes. Thus any pair of features might have a number of genes in common, but the leaves of the tree are expected to have very little overlap. The overlap statistics prior to, and post, making the tree are highlighted in the lower and upper triangles of the Figure **2C**, respectively. It is very clear from the heat-map that the nuclear feature hits segregate into a separate set even prior to making the tree; but there is a clear structure to the way genes affect endocytic features. In the context of the tree, this structure is visible as connected nodes and leaves, termed fluid (10–15; the F-features) and Transferrin (16–24; the T- and Okt-features) nodes, the endo-common node (3–9), and the nuclear node (1,2, 25, 26; Nuc-features). These nodes have high bootstrap support, and represent robust aspects of the underlying data (Figure **S5**).

### Independent support for the tree structure

The tree by definition organizes genes and features into a hierarchy. However, there are several independent lines of evidence supporting this hierarchical organization: 1) Pairs of leaves are significantly depleted in the number of overlapping genes. This would not happen if genes affected features at random; for example, a gene affecting a single fluid feature and a single Tf feature would not migrate up the tree, but rather would remain stuck at the two leaves. There is one interesting exception: the Fdex and Tf colocalization leaves (Fclc and Tclc) share a high proportion of genes, as would be expected since two-way colocalization between endosomal probes is highly correlated (Figure **2C**). 2) Several nodes are populated by genes that have similar predicted function, and a certain subset of nodes are enriched for protein-protein interactions within themselves (Figure **S4A**) (as assigned by STRING, http://string-db.org/ [29]); a complete set of interactions for the nodes enriched for within-node interactions is listed in Figure **S4B**). 3) Genes with previously established functions in endocytosis are found in nodes consistent with the endocytic features they are expected to affect. For instance, genes known to affect CD endocytosis remain in the nodes of the tree that represent Tf uptake features (Table **S2**).

Node 3 (the root node; Figure **2A**) is the shared bifurcation point above three main branches – and corresponds to genes potentially affecting endocytic pathways, cell size and nuclear morphology features. This node has only one gene (Table **S2**; CG12498 or *zasp52*), which influences cell size and endocytic features but not nuclear features. This indicates a clear segregation of genetic control among the three branches. Interestingly, genes that affect nuclear morphology are significantly correlated with higher cell proliferation, while those that affect endocytosis typically reduce cell number [22].

The tree structure also provides evidence for two categories of genes that affect endocytic processes: 1) Genes that constitute a common machinery between the two pathways populating the nodes 4–8 (Figure **2A**: Table **S2**). 2) Genes that separately affect the fluid-phase (nodes 10–15) and Tf (nodes 16–22) pathways. Notably, genes involved in proteasome, ribosome and spliceosome function are almost all located at nodes 4–7 (see Table **S2**). It is probable that these housekeeping genes are represented here due to their pleiotropic nature, resulting from the broad range of proteins and mRNAs that could be affected by their perturbation. However, their presence is nevertheless significant since genes at nodes 4–7 do not affect nuclear morphology features, and may represent a more general layer of control of endocytic processes.

### GO annotation of the tree nodes

We have statistically established that our screen has a high false negative rate (∼30%, Figure **S2B**). This means that some genes might be missed when examining any individual feature. However, it is unlikely that a protein complex of many components will be missed in its entirety. To explore this, we replaced the gene IDs with their Gene Ontology (GO) ‘cellular component’ annotation at the tree leaves (Table S3). The analysis was then repeated as before, except that it was GO annotations rather than individual genes which were allowed to migrate to higher nodes of the tree. This process was useful for two distinct reasons.

First, as expected, protein complexes became visible even when some of their components might have been missed at individual leaves. For example, components of clathrin adaptors and vesicle coats were individually close to the leaves of the original tree, but the clathrin and coat complexes themselves rose to nodes 17 and 22, which head the genes influencing the CD pathway (Figure **2B**).

Second: common cellular machinery such as the proteosome, splicesome and ribosome rose to nodes close to the tree root (node 4 and 6), indicating that these protein complexes had pleiotropic effects across almost all features (Figure **2B**; Table **S3**). This enabled us to objectively separate housekeeping genes from genes of specific interest in endocytosis.

Of particular interest to us was the observation that genes affecting several Tf intensity and morphology features converged at certain nodes. The umbrella node for the CG-specific annotations is node 10, and is populated by, amongst other genes, the phosphoinositide-3-kinase (PI3K): *pi3k68d* (Figure **2B**). The closest human homolog of *pi3k68d*– PI3KC2α– was recently confirmed to be a key regulator of dynamin independent endocytosis [30]. Node 10 also contains two genes likely to function in endosomal pathways: *dVps26* (homolog of a component of the retromer complex important for endosome to Golgi trafficking [31]); and *cg1515*, (a *Ykt6* like SNAP receptor protein implicated in multiple fusion steps at the Golgi and endomembranes [32]).

### A secondary classification assay

**Figure 3:**
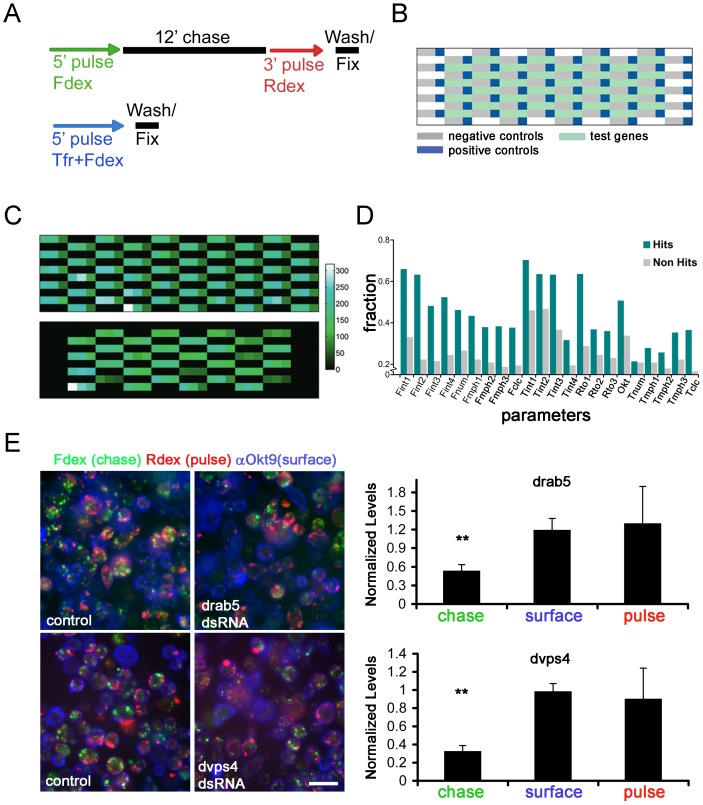
Primary hits validated in a secondary classification assay. (A–B) Schema (A) and positional patterning (B) on cell arrays of secondary endocytic classification assays carried out for all CG features (upper schema) or a subset of CD features (lower schema).All the test genes were surrounded with local positive controls, and negative controls (see legend in (B)). With this patterning, each gene was tested in triplicate, with three local positive controls and six local negative controls. (C) Heatmap representing raw mean fluorescence intensities (in the pulse channel) across a test cell array used to validate the CG secondary endocytic assay described in (A). Only the means of control wells are shown in the top panel and the inter-control variation in means is representative of a typical experiment. For comparison, the lower panel depicts the mean fluorescence intensities of test genes. (D) The green bars show the fraction of genes predicted as hits for each feature in the primary screen that were also picked up as hits for that feature in the secondary. The gray bars show the fraction of genes not predicted as a hit for each feature in the primary screen that were nevertheless picked up as hits for that feature in the secondary. With a single exception (Tnum) we find that the green bars exceed the gray (p-value 5×10^−6^ for 22 fair coin flips) demonstrating the selectivity and reproducibility of our primary assay. (E) Psuedocoloured fluorescence micrographs of representative control and *drab5*- and *dvps4*- dsRNA treated populations of cells that were subjected to the CG pulse-chase assay from (**A**). Both Drab5 and Dvps4 depleted cells were affected in the chase (with Fdex, green) portion of the assay, while the pulse portion (with Rdex, red) was unaffected (see quantitation in bar graphs on the right, normalized to control). Scale bar = 10 µm.

The gene PVs provide detailed information across our assayed features, and the tree-based clustering allowed broad segregation of genetic modules according to the features they affect. To isolate endocytic gene modules with higher resolution, selected genes with significant PVs were assayed for the CD and CG pathways in a modified cell array format. Three RNAi test wells were surrounded by positive and negative controls to mitigate positional artifacts, and assayed at different pulse points (Figure **3A, B, C**). Though this is a low-density format, it allows us to avoid the normalization procedure, and therefore to find the direction of any effect induced by RNAi.

We selected 316 genes that affected Tf features and tested them with a short pulse of Alexa 568-Tf/Fdex for 5 minutes (Figure **3A**). 245 (∼80%) of these genes were observed to influence Tf uptake. For all the 470 genes that influenced CG features in the primary, we pulsed F-Dex for 5 minutes followed by a chase of 12 minutes and a second pulse of another fluid-phase probe (TMR-Dex) for an additional 3 minutes (Figure **3A**; upper scheme). This would allow classification of genes according to an early (pulse) or late (chase) effect in the pathway. Of the 470 genes tested at higher time resolution, 431 (92%) showed phenotypes in the secondary characterization for fluid features. For both pathways, genes were observed to influence individual features in the secondary assay as predicted by the primary assay (Figure **3D**). This provided strong evidence of the power of our shape-based strategy to detect hits.

**Figure 4:**
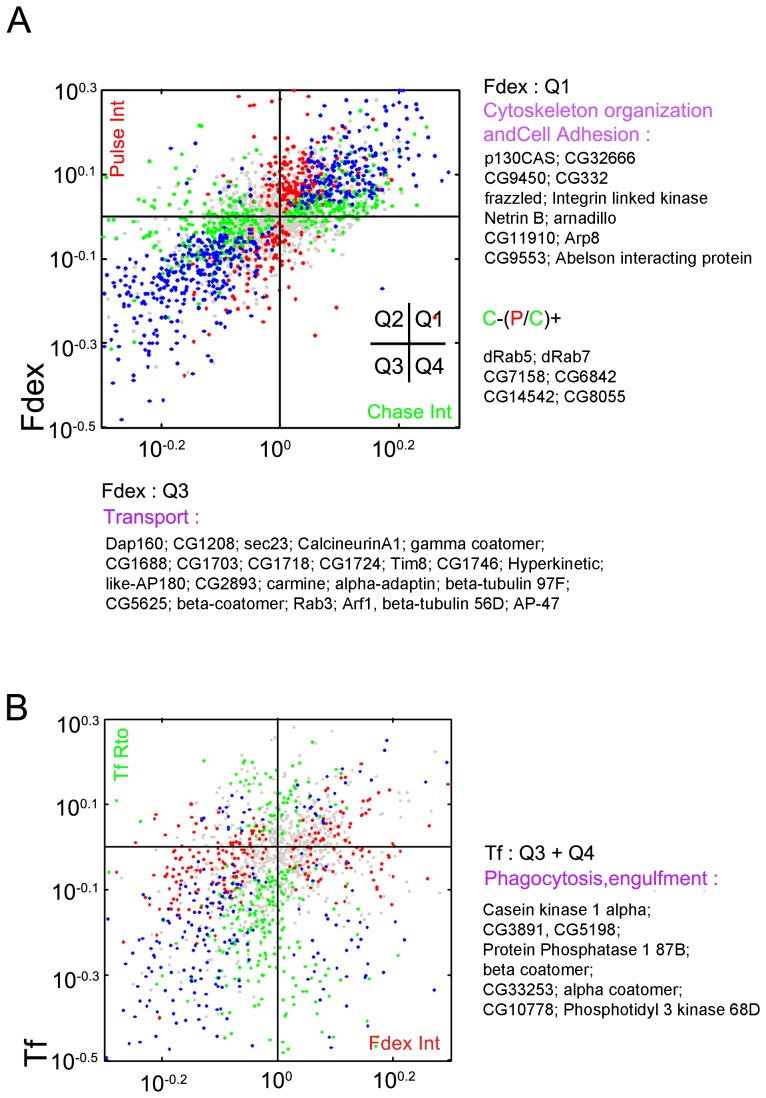
An interpretive summary of classified genes. (A) Normalized ratios (with respect to local controls) of the fluid-phase pulse intensity vs. chase intensity features for all test genes from the classification assay. See inset axes for quadrant numbering. Quadrant color map:green, genes with only chase features increased or decreased; red, genes with only pulse features increased or decreased; blue, genes with both pulse and chase features increased or decreased; gray, genes that did not significantly perturb these features. A selection of GO categories and genes from quadrants 1 and 3 have been highlighted for the fluid pulse vs chase features; the remaining GO categories and genes populating all quadrants are listed in Table S4. Similar quadrant analyses (not shown) were performed for chase intensity vs. pulse-to-chase ratio (Table S4). Genes involved in lysosomal routing, multivesicular body (MVB) sorting and membrane deformation emerged in quadrant 2 (C−, P/C+). (B) Normalized ratios (with respect to local controls) of the TfR ratio vs fluid intensity features for all test genes from the classification assay. Quadrant color map: red, genes with only fluid features increased or decreased; green, genes with only TfR features increased or decreased; blue, genes with both fluid or TfR features increased or decreased; gray, genes that did not significantly perturb these features. Transport genes collected from quadrants 3 and 4 are highlighted (right), and the rest of the GO categories and genes populating populating all the quadrants are listed in Table S4.

The additional information provided by the secondary assay (intensity/morphology values combined with pulse up/down and chase up/down) were useful in segregating genes within the CG specific nodes (Figure **4A**). Interestingly, when placed into a quadrant plot, the majority of genes fell into quadrants corresponding to two broad categories – those that either increased pulse and increased chase (quadrant 1; 142, or decreased pulse and decreased chase (quadrant 3; 185), with a minority showing differing effects on pulse vs. chase (quadrants 2 and 4; 28 and 64, respectively).

Quadrant 1 contains the genes in the CG pathway whose absence causes accumulation of both pulse and chase probes in the cell. Based on predicted (from homology) or known interactions, several quadrant 1 genes form a network that is enriched in actin-related functions such as cell adhesion and cytoskeleton-membrane linkage. Prominent members include *chickadee* (profilin), actin related protein 8 (*arp8*) and Abelson interacting protein (*abi*). The presence of focal adhesion regulators integrin linked kinase (*ilk*) and, *p130Cas* [33] in this quadrant suggest that this network, acting to regulate membrane-cytoskeletal linkages or tension, negatively regulates endocytosis [34]–[36].

Quadrant 2 contains genes that affect the chase portion of the fluid uptake route, but not the early short pulse, thus placing them later in the pathway. This quadrant contains *drab5*, which is required for homotypic and heterotypic fusion between early and late endosomes; Table **S4**). Since Drab5 is not found on GEECs [8], [11], and it regulates cargo trafficking from early endosomes, its absence leads to an accumulation of pulse and reduces the fraction of cargo reaching late endosomes (chase), resulting in a high pulse:chase intensity ratio per cell (Figure **3E**). Similarly, other components required for routing to the lysosome such as *drab7* and *CG7158* (the fly homolog of the Rab5GEF ALS2), as well as three conserved ESCRT III complex proteins (Dvps4 (CG6842; Figure **3E**), Dvps2 (CG14542) and Dvps32 (CG8055), whose homologs are required for multivesicular body (MVB) sorting and membrane deformation [37], [38]) all appear in this quadrant (Table **S4**).

Quadrant 3 consists of genes whose absence causes a reduction in both pulse and chase signal, and these may be most directly involved in the CG pathway. It is the largest quadrant, and consists of three main predicted GO classes: a prominent group representing proteasomal machinery; a subgroup of genes that affect mitotic spindle organization; and a third group of genes whose GO terms predict functions in endocytosis and phagocytosis. Notably, most of the genes from the proteasomal and mitotic spindle groups also affect the CD pathway, so are not exclusive to the CG pathway (Table **S4**). Within the third group, there is a specific cluster of genes that encode the vesicle coat proteins α-COP, β-COP, Arf1, and Sec23, which also affect Tf features. Just peripheral to this latter cluster are genes such as *pi3k68d*, the vacuolar ATPase E subunit, *vha26*, and the fly homolog of VPS33A, *carnation* (an SM protein), which specifically block only the CG pathway (see below).

In the context of the CD pathway (Figure **4B**), genes in quadrants 3 and 4 (genes required for normal levels of Tf uptake) include expected components of the clathrin coat such as *dap160* (dynamin associated protein 160), *lap* (like AP-180), *alpha-adaptin* and *ap-47*. Other members of these quadrants include several *beta tubulin* genes (56D, 85D and 97EF), *pp2A-29B* (protein phosphatase 2 subunit A) and *canA1* (Calcineurin A1 subunit). These were also identified recently as important for TfR uptake in mammalian cells [39].

Since the tree structure indicated the presence of common machinery for the CD and CG pathways, we examined the intersection of the two gene sets obtained from the classification assay. We found ∼100 genes whose absence causes a reduction (short pulse) in CG pathway uptake and also affects CD endocytosis negatively. This set is enriched in components of the proteasomal machinery, genes required for cell proliferation, and those required for response to cell stress (Figure **S6A**).These processes are likely to comprise the basic peripheral cellular requirements for these two major endocytic routes to operate efficiently.

Interestingly, it was only a small fraction (<1%) of genes with phenotypes in the classification assay whose absence was found to increase the output of both pathways (Table **S4**; Figure **4A**, quadrant1). An interesting candidate here is integrin linked kinase (*ilk*), which negatively regulates both CD and CG endocytosis, and is known to link the cytoskeleton to the plasma membrane during matrix adhesion [40]. *ilk* was different from other cortical actin regulators such as *abi* and *chickadee*, which were required to augment solely the CG pathway. Notably the depletion of Ilk caused a reduction in cell size, and this was also seen in the cortical actin/linkage regulators such as dTalin (*rhea*) and Formin like protein (CG3213*8*). Both of the latter two genes are known to affect cell shape/size [41], but do not affect endocytosis; they populate the ‘cell size’ node in the tree structure. Hence Ilk-mediated matrix adhesion may have a distinct role as negative regulator of both CD and CG endocytosis.

Finally, there is a group of genes were those whose depletion had opposing effects on CG versus CD uptake (Table **S4**, Fluid Intensity Quadrants, Quadrant 2). The depletion of dRab7, and the Rho GTPases, RhoBTB and RhoGAP102A, as well as the actin-nucleating formin Diaphanous, caused reduced CG uptake while increasing CD endocytosis. We decided to examine more closely the role of genes annotated broadly to the cytoskeletal sub-group by utilizing the pulse chase classification assay in Figure **4A** (Table **S7**, Figure **S6B**). The presence of proteins that form a subset of actin filament remodeling activities (Slingshot, activates cofilin; [42]), Coronin (Arp2/3 regulatory activity [43]), Arpc1 (suppressor of Profilin), Twinfilin, Capping protein [44]) again implicates a central role for a dynamic actin remodeling pathway in CG endocytosis. We also note here that the depletion of dFak, the *Drosophila* ortholog of the focal adhesion kinase FAK [45], affected the CG pathway similarly to the focal adhesion regulators previously discussed (p130Cas, Ilk). Further links between the CG pathway and cell adhesion were revealed by a subset of laminin-related genes whose depletion specifically affected CG endocytosis – the netrin receptors *fra* (Frazzled), *sli* (Slit), *unc-5* (dUnc5), and the netrin ligand *NetB* (NetB) (Figure **S6B**). Netrins and their receptors modulate cell-matrix interactions in diverse cell types and thereby affect focal adhesion pathways [46].

### Comparison of RNAi perturbations and fly mutants

**Figure 5:**
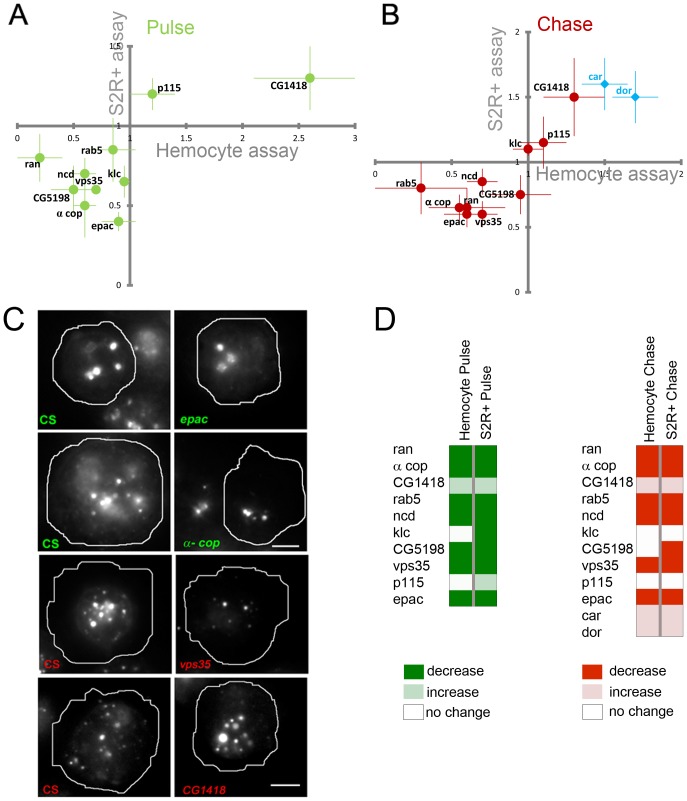
Endocytic phenotypes in mutant primary hemocytes from *Drosophila*. (A–D) dsRNA treated S2R+ cells phenocopy corresponding allelic mutants in primary hemocyte cultures in a secondary assay. Scatter plots (A, B) show normalized fold change in fluorescence intensity of dextran that was pulsed (A) or chased (B) in S2R+ cells treated with different dsRNAs (y axis) or in hemocytes (x axis) from the corresponding mutant flies. In all cases, representative values were normalized to those from negative controls (CS hemocytes or zeo dsRNA treated S2R+ cells) and are plotted as mean± SEM. (n>30 for hemocyte assays, n>200 for S2R+ assays in all cases). For the chase assay in (B), we utilized *dor^4^* and *car^1^* mutant hemocytes as positive controls (shown in light blue; Sriram et al., 2003). (C) Representative micrographs of hemocyte cultures from flies carrying hypomorphic alleles of *vps35*, *epac*, *α-cop* and *CG1418* assayed as in (B). (D) Summary of the experiment in (A–B) displaying statistically significant (Student's T-test, p<0.05) changes in uptake/retention of mutant hemocytes or gene-depleted S2R+ cells as colour coded maps. Scale bar in (C) = 5 µm.

We next tested endocytosis in primary cells derived from fly lines carrying mutations in each of a set of 13 genes (Table **S5**). Four of these mutations resulted in lethality before the third instar larval stage, suggesting important roles in development, while 10 gave viable third instar larvae from which we could culture hemocytes. We compared the fold change in uptake in the hemocyte and S2R+ pulse-chase assay for these mutants and their corresponding gene depletions with dsRNAs (Figure **5A, B**). For the hemocyte assays, we used primary cells from wild type CS flies and from known late endosomal mutants (*dor^4^*, *car^1^*; [47]) as chase controls (Figure **S7C**). Overall, in 11 out of the 12 genes tested in the chase assay, we found a similar pattern in the perturbation of CG endocytosis in hemocytes (Figure **5D**) compared to those determined in S2R+ cells when depleted of the respective gene products. Similarly, 8 of 10 genes gave similar trends in the pulse assay between S2R+ and hemocyte experiments (Figure **5D**). We also tested endocytic uptake in hemocytes from two fly lines carrying mutations in genes that did not score in the primary dsRNA screen; mutants of *drp* and *flotillin* did not show any measurable defects in endocytosis of the fluid phase (Figure **S7A, B**). These results support the idea that our dsRNA-based analysis provides a reliable and robust method and resource for exploration of specific gene targets involved in endocytic pathways.

### New gene functions in the CG pathway

We explored two components whose identities could not have been predicted from available knowledge of the genes involved in endocytosis. These are located in quadrant 3, a Sec/Munc protein, Carnation, and the Vacuolar ATPase subunits, identified as specific regulators of endocytosis via the CG pathway.

#### Role of a Sec/Munc protein complex in CG pathway

**Figure 6:**
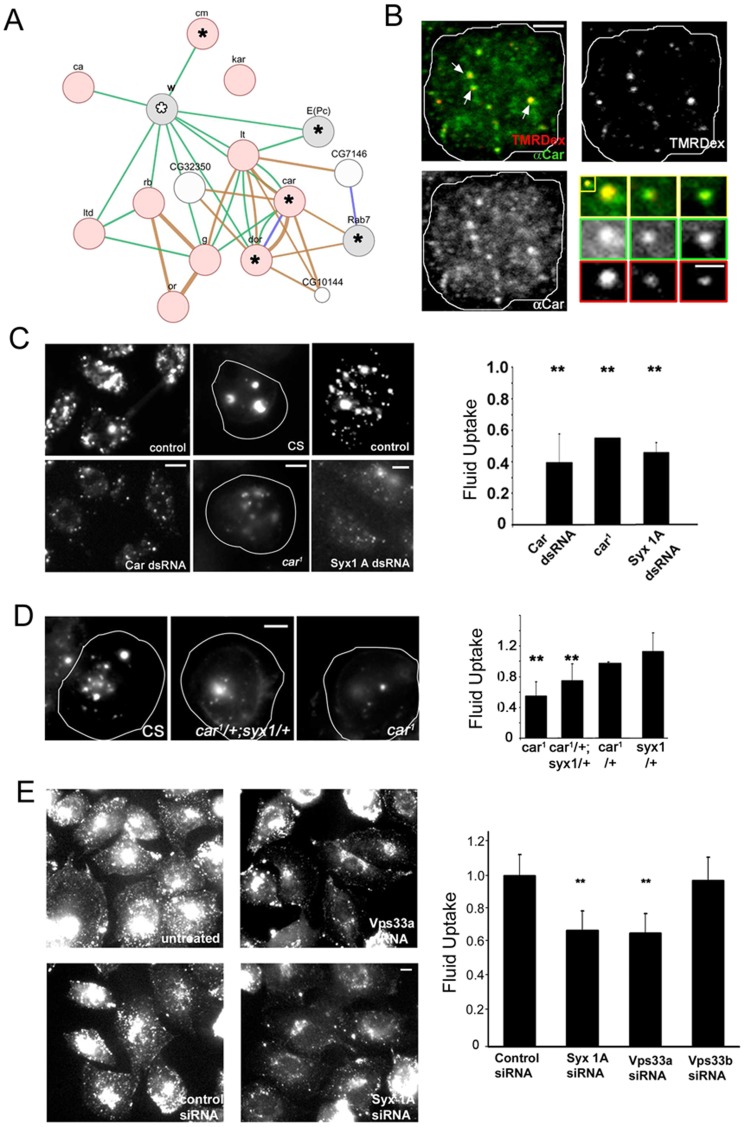
Role of lysosomal genes. (A) Network map depicting known and predicted interactions (green lines: genetic; blue lines: physical; brown lines: predicted based on conserved data) between the ‘Granule group’ set of eye colour mutants (pink) and selected hits (gray). In this network, genes encoding Carnation (*car*; the fly homolog of VPS33), Deep orange (*dor*), Carmine (*cm*) and Rab7 were identified with roles in CG endocytosis in this study (denoted by black asterisks), while White (*w*) depletion affected at least one Tf pathway feature (white asterisk). (B) Localization of Carnation on early fluid endosomes. *Drosophila* S2R+ cells were pulsed with TMR-Dextran for two minutes and fixed and labeled with antibodies to Carnation (αCar). Micrographs show a representative cell imaged in two channels and a pseudo colour merge image (labeled TMRdex and αCar), in red, green and merge respectively). Carnation (green) is seen enriched on peripheral, small, early fluid endosomes (red). Three examples of such endosomes (white arrows in merge panel) are shown in the magnified inset. (C) Fluorescent micrographs depict the levels of fluid uptake in representative S2R+ cells treated with dsRNA against *car* (first lower panel) or *syx1A* (last lower panel) or in hemocytes from *car^1^* mutant flies (middle lower panel), with their respective controls (upper panels). Bar graph represents mean and SD of normalized fluorescent integrated intensity per cell from 2–3 experiments, with 100–150 cells per treatment (S2R+ cells) or 40 cells per genotype (hemocytes). (D) Representative fluorescent micrographs depict fluid uptake measured in hemocytes as in (C), in flies that were: homozygous for a mutant allele of *car* (*car^1^*); a hetero-allelic combination of *car^1^*/+;*syx1*/+;or wild type (CS). Also tested were flies heterozygous for *syx1*/+ and *car^1^*/+. Bar graph represents mean and SD of normalized fluorescent integrated intensity per hemocyte from 2–3 experiments with 40 cells per genotype. (E) Representative micrographs show human AGS cells treated with control siRNA or siRNA to hSYX1A and hVPS33A/B and pulsed with FITC-Dextran for 5 min. Right panel - Bar graphs show population averaged mean fluorescence intensity uptake per cell (representative experiment with n>50 cells per replicate, 2 replicates). Scale bar in (B–E) main panel = 5 µm, inset = 1 µm. Double asterisks denote significance *p* values lower than 0.01 with the Student's T-Test.

Carnation (Vps33) is a component of the HOPS tethering complex, a downstream effector of Rab7 required for late endosomal delivery of Golgi proteins and lysosomal biogenesis [48]. While Car is known to affect a late trafficking step in endocytosis of fluid-phase or receptor cargo (together with Dor [47]), a role in early uptake via the CG pathway was unexpected. We explored the effect of depleting Car on the CD pathway. There was no change in normalized Tf uptake (Figure **S8A**) despite strong inhibition of the CG pathway in the same cells (Figure **6C**). Furthermore, in hemocytes derived from *car^1^* mutants, a decrease in fluid phase uptake was observed compared to the wild type control (CS) (Figure **6C**) while endocytosis via the CD-pathway (as monitored by a Cy3-labeled maleylated BSA, a ligand for CD uptake in hemocytes; [7]) was unaffected (Figure **S8B**). While Car has been previously localized to late endosomes [47], here we show that Car also localizes to fluid-phase endosomes much before they fuse with cargo from the CD pathway (Figure **6B**).

Car belongs to the Sec1p/Munc18 (SM) family of proteins [49], which regulate the efficiency and specificity of membrane fusion events through their interactions with SNAREs [50]. Car has been shown to interact with the lysosomal SNARE, syntaxin 16 [51] and regulate fusion of late endosomes with lysosomes [47]. We reasoned that its function at early fluid endosomes is also likely to be mediated via regulation of SNAREs. Since Car does not interact with a characterized early endosome SNARE [51], we explored if Car might interact with the plasma membrane SNARE, Syntaxin1A (Syx1), and whether this interaction was important for CG endocytosis. We find that Car and Syx1 do interact genetically: a hetero-allelic combination (where 1 copy each of Car and Syx1 are mutated) caused a decrease in fluid uptake (Figure **6D**), although single-copy mutations of either one alone did not inhibit fluid phase uptake (Figure **6D**). Furthermore, depletion of Syx1 by RNAi causes a specific decrease in fluid uptake (Figure **6C**), but does not affect normalized Tf uptake (Figure **S8C**).Thus, using mutants as well as RNAi-based depletion we show that both Car and Syx1 specifically affect the CG pathway in *Drosophila* cells. We then examined how Car modulates Syx1. We found that the level of Syx1A was significantly decreased in Car-depleted cells compared to control cells (Figure **S8D,E**). Thus Car, like the SM protein family member Munc18-1 [52], could directly regulate Syx1A stability in these cells.

We next tested if these newly identified components are conserved in their function in vertebrates. We find that depleting the human homologs of Car and Syx1 i.e., hVps33a and hSyx1, also results in inhibition of CG but not CD endocytosis in a human cell line (AGS; Figure **6E** and Figure **S8F**). While Vps33a specifically affects the process, depletion of Vps33b did not perturb fluid uptake. Thus, we have identified novel, conserved functions in CG endocytosis for two genes with no previously characterized link to the pathway.

#### Role of Vacuolar ATPase (V-ATPase) complex

**Figure 7:**
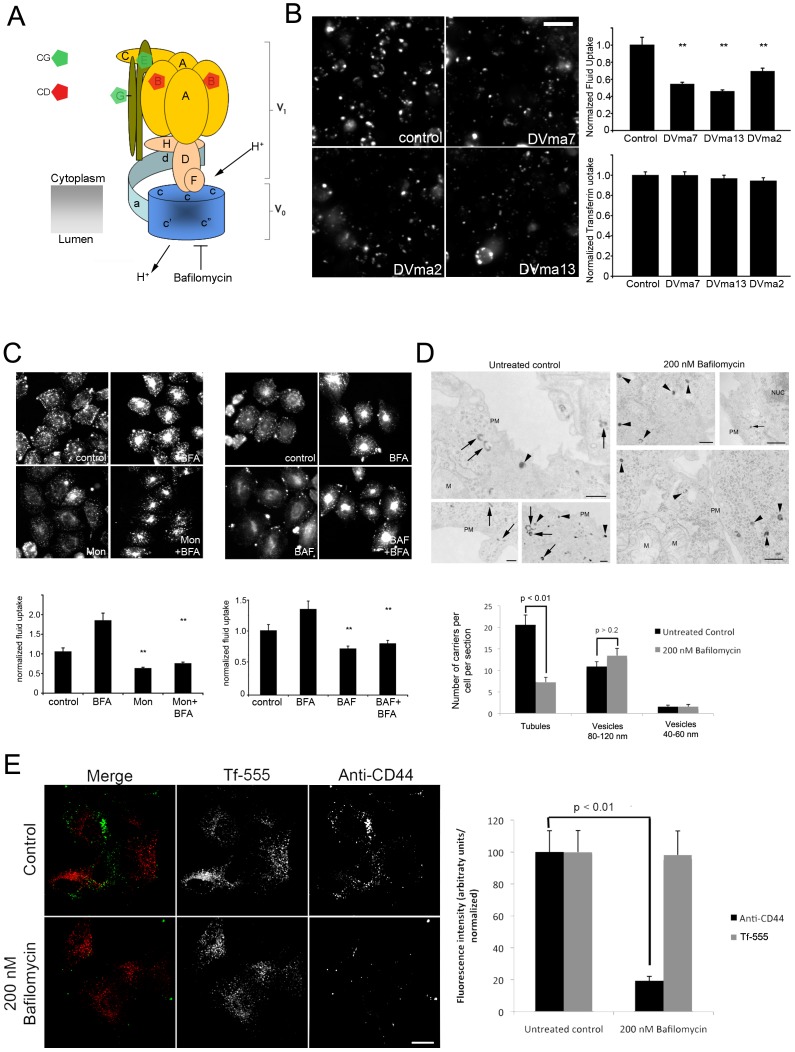
Vacuolar acidification and endocytic regulation. (A) Cartoon depicting arrangement of components of the V-ATPase complex [76] in *Drosophila*, identified for their involvement in the CG (green) and CD (red) pathways in the primary screen. (B) Fluorescent images show representative micrographs of S2R+ cells treated with control dsRNA or dsRNA against V-ATPase V1 subunits F (*dvma7*), B (*dvma2*) and H (*dvma13*).Bar graphs on the right shows normalized FITC-Dextran uptake (fluorescent integrated intensity per cell; upper panel) or normalized Tf uptake in the same cells (calculated as amount of internalized Tf normalized to cell surface Tf receptor; lower panel).Values are mean ± SEM from a representative experiment with n>100 cells per replicate, 2 replicates). (C) CHO cells were either untreated (control) or treated with Monensin (Mon; left panels) or Bafilomycin A (right panels) in the presence of Brefeldin A (BFA) or absence, and then assayed for fluid uptake with a pulse of TMR-Dextran (Methods S1).Fluorescent micrographs show representative fields of cells after the corresponding treatments (indicated in white). Bar graphs depict data from a representative experiment showing mean fluorescent integrated intensity per cell for each condition, normalized to untreated controls (mean ± SEM, of 2 replicates, n>50 cells per replicate). Double asterisks denote significance *p* values lower than 0.01 with the Student's T-Test. Scale bar in (B) = 10 µm, (A) = 20 µm. (D) Untreated control MEFs or those treated with Bafilomycin A were pulsed with HRP for 2 min and then fixed and processed for EM (Methods S1). EM micrographs were counted for the presence of small (40–60 nm) or large (80–120 nm) vesicles (marked by black arrowheads) or the more complex early tubules that correspond to the CG pathway (marked by black arrows). Bar graph shows averaged data from 4–6 cells per experiment across 2 independent experiments, *p* values are indicated for comparison sets using Student's T-Test. Scale bar = 200 nm; M = mitochondrion; PM = plasma membrane; NUC = nucleus. (E) MEFs were treated with Bafilomycin A and assayed for CD44 and Tf uptake as described [9], [13]. Fluorescent micrographs show representative fields of cells. Bar graph depicts data from three experiments showing mean fluorescent integrated intensity per cell for each condition, normalized to untreated controls (mean ± SEM, of 3 replicates, 10–12 cells per replicate). *p* values are indicated for comparison sets using Student's T-Test. Scale bar = 10 µm.

The V-ATPase annotation at node 9 (the bifurcation point for the two pathways; Table **S1**), and the locations of the individual V-ATPase subunits in the common CG and CD pathway nodes, suggest an important role for the V-ATPase in both the endocytic pathways. While acidification and its role in the CD endocytic pathway is well established during later stages of endocytosis in ligand dissociation as well as in traffic through the early and late endosomal pathway [53], [54], its role in CG endocytosis is unclear. Notably, GEECs achieve a very low pH (∼6), within minutes after formation [11], and since we identified the V-ATPase E subunit (fly ortholog *vha26*) as an early regulator of fluid phase uptake, we examined other selected components of the V1 V-ATPase complex for a similar effect (Figure **7A**). Depletion of subunits F (Vha14; DVma7), B (Vha55; DVma2) and H (VhaSFD; DVma13) inhibited CG, but did not affect CD endocytosis (measured as the ratio of Tf internalized to TfR surface levels, although surface levels of TfR and Tf uptake are also affected; Figure **7B**).

To rule out indirect effects of the chronic inhibition of V-ATPase, we used an inhibitor of the Na+/H+ anti-porter, Monensin [55] as well as Bafilomycin A1, a specific V-ATPase inhibitor [56] to rapidly alter pH in the endolysosomal system in mammalian cells. In both cases, treatment with 50 µM Monensin or 250 nM Bafilomycin A caused a significant decrease in fluid-phase dextran uptake in CHO (Figure **7C**) and S2R+ (data not shown) cells within minutes of application. This effect was independent of secretory pathway function in mammalian cells; both Monensin and Bafilomycin reversed the surge of fluid phase uptake observed after addition of Brefeldin A (Figure **7C**), which is a potent inhibitor of secretion and generates excess available Arf1 after release from Golgi membranes for CG endocytosis [8], [14].

To address the issue whether the role of vacuolar ATPases in CG endocytosis is a general requirement of CG endocytosis, we assessed whether Bafilomycin affected the endocytosis of both fluid-phase and membrane cargo in a mammalian cell line. Bafilomycin significantly affects the uptake of HRP (Figure **7D**), and notably CD44 (Figure **7E**), as well as causing a drastic reduction in the generation of early tubular CLICs, while leaving the later, more vesicular carriers unaffected (Figure **7D**; [9], [13]).

These results support a conserved requirement of V-ATPase function in endocytosis via the CG pathway.

## Discussion

### Exploiting cell-to-cell variation to screen for genes involved in endocytosis

The single cell approach that we have outlined here is likely to be generally applicable for investigating cellular processes that are by nature prone to inter-cell variability. We have demonstrated the viability of this approach in the context of a systematic examination of the conserved *Drosophila* genome to identify genes involved in CG and CD endocytosis. Many of the genes that we have identified segregate to distinct endocytic or morphological classes, and several classes contain functionally related proteins which show high connectivity. These classes potentially link diverse processes, such as protein synthesis and degradation, cell proliferation, membrane trafficking, focal adhesion and cortical actin dynamics with CG endocytosis. Based on the design of our study, many identified components with roles in endocytosis are also likely to be highly conserved across species.

Quantitative assessment of the false positive and negative rates of the screening methodology, based on a statistical analysis of the distribution of Z-scores of the individual genes, makes the results of this genome-wide screen a reliable resource for further studies. In particular, the low (<10%) false positive rate implies that about half the genes we report as hits are true positives (<700 genes of the total 7109 will emerge as false positives for any given feature). At the same time, it should be highlighted that the screening methodology adopted here has a relatively high overall false negative rate for a given feature (∼30%; Figure **S2B**). False negative rates are rarely estimated in genome-wide studies, partly because most primary screens are carried out without multiple biological replicates, and without a large number of positive and negative controls. It is in fact quite likely that false negative rates in many primary screens are fairly high, given that independent screens targeting the same signaling pathway often identify very different gene sets [57], [58].This is highly reminiscent of the poor overlaps between multiple mass spectrometric screens for identification of specific protein complexes, an effect now attributed to different means of preparing samples as well as a lack of an empirical framework for their analysis of false negatives [59].

### Endocytic modules

**Figure 8:**
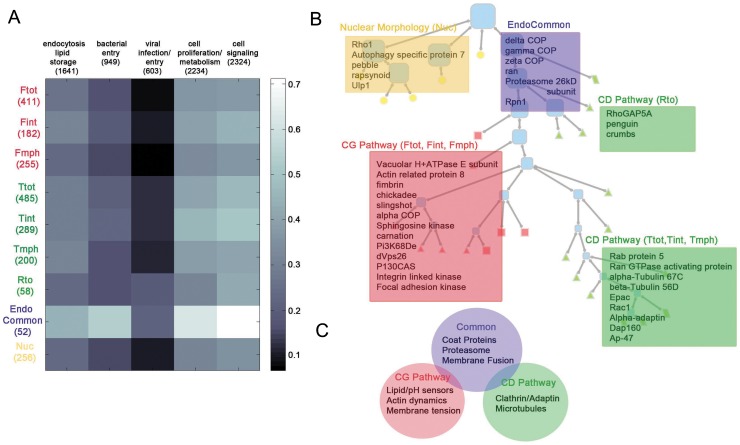
Relationships of hit subsets to Genome wide screens and an interpretive summary of primary hits. (A) Relationships of hit subsets to other screens. The heat map shows the degree of overlap between hits in different tree categories (rows) with various other RNAi screens (columns). Overlap is quantified as the fraction of genes in a given tree category also identified as a hit by the group of screens. The number of genes in each grouping is denoted in brackets. The screens are grouped as shown in Table S8, first tab. (B) Selected genes from collected nodes (representing the CG pathway (red box), CD pathway (green boxes) and nuclear morphology (light orange box) parameters) are shown overlaid onto the tree hierarchy.

Building trees to represent multi-feature biological data is likely to be a generally useful approach over traditional distance-based hierarchical clustering methods [60]–[62]. The tree-based method provides a direct way of identifying functional modules and their molecular basis. The genes identified here with roles in CG and CD pathways show different degrees of overlap with genes identified for other cellular processes in other genome-wide screens (Figure **8A**; Table **S8**).Intriguingly, members of the root nodes 2–3 and the endocytic nodes 6–9 (but not the more pathway-specific nodes) are commonly identified in bacterial entry/infection screens (Table **S6**). These general observations reflect a tendency for bacteria to hijack internalization routes into the cell in an opportunistic fashion. Furthermore, genes from the common endocytic nodes are also highly represented in screens that assay cell proliferation and metabolic pathways, and those that involve cell signaling (Figure **8A**). These pathways require genes that affect endosome number and morphology features, and to a lesser extent, endosomal intensity. This is internally consistent with our own assays, which indicate a dependence on the same features for cell proliferation [22].The overall low level of overlap with genes that affect viral infection and entry is also interesting, and may reflect a divergence in host virulence factors given the evolutionary distance between metazoans [63].

When overlap with selected genome-wide studies was examined, some clear patterns emerged. We find that the Arf1/COP1 complex members are necessary for the CG pathway and several of these are also necessary for lipid droplet formation [57] and for Rho GTPase localization to the plasma membrane at sites of SOPE injection by *Salmonella typhimurium -* effects that have been ascribed to a central role of COPs in cholesterol homeostasis [64]. Given that CG endocytosis requires Cdc42 activation at the cell surface via a cholesterol-sensitive mechanism [12], it is likely that the requirement for COP1 in CG endocytosis may reflect its ability to regulate cholesterol distribution in cells. Indeed, replenishing cholesterol in a COP1-mutant cell line restored endocytic uptake, whereas a similar treatment with cholesterol had no restorative effect on Arf1 knock down cells (Thottacherry and Mayor, unpublished results). Alternatively, COPs could have a direct role in shaping endocytic invagination at the cell surface.

While the role of actin regulation in CG uptake is well established [8], [10], [12], the identity of the responsible molecular machinery is largely unknown. Here we provide endocytic signatures (Figure **8B**, Figure **S6B**, Table **S7**) of several conserved cytoskeletal components and identify several candidates for further study. The PVs of Profilin, Abelson interacting protein (*abi*) and the formin *diaphanous* and actin remodeling agents, Unc-5, Fimbrin as well as Twinfilin, indicate important roles in CG endocytosis, while the membrane tension regulators and transducers (*ilk* and *p130Cas*) also segregate with negative regulatory control in the CG pathway. Some of these candidates (Table **S7**) also affect CD endocytosis, consistent with earlier evidence in mammalian cells [65], [66].This supports the idea that the CG pathway is exceedingly sensitive to the physical state of the cell membrane, namely tension, lipid availability and actin modulatory capacity. The enrichment of Tf-uptake features in tubulin genes PVs suggests a contrasting dependency on the microtubule skeleton for CD endocytosis (Figure **8B**).

We also identify a number of signal transduction and kinase cascade families within our hit-set (Table **S2**), with evidence for the functional segregation of kinases for different pathways of endocytosis [67]. The presence of many membrane-associated proteins encoded by genes that affected the two pathways (see Table **S2**) also points to regulatory control of the two pathways by *cis*-acting elements in the plasma membrane. An example of such control is observed with the axon guidance genes, where down-regulation of the *frazzled* receptor or its soluble ligands *netB* and *unc-5* act to enhance CG-pathway activity (Figure **S6B**).

### New molecular players involved in CG endocytosis

We have discovered new components of the endocytic machinery, thus validating the high-throughput genome-wide design of our study. The identification of a role for the SM protein Car/hVPS33A in the CG pathway, along with a SNARE counterpart (Syx1), suggests that the CG pathway may be sensitive to regulation at the level of the availability of SNAREs.SM proteins have been previously shown to modulate levels of Syx1 [52], [68]. Subsequently, Syx1 may be required for the normal delivery of some component that is critical to CG endocytosis. Alternatively, the levels of Syx1 itself could be a direct sensor for the functioning of this pathway, since activated Cdc42 may directly interact with SYX1A at the cell surface [69]. Interestingly, Vps45 (CG8228), another SM protein in *Drosophila*, specifically perturbs CD endocytosis (Table **S2**), in our study. Thus the two SM proteins Car and Vps45 may serve to regulate the CG and CD endocytosis, respectively.

In *Drosophila*, Car belongs to a special class of eye color genes, called the “Granule Group” [70]. Several members of this group, in addition to affecting pigment granule biogenesis, also affect multiple steps of intracellular traffic and are components of the HOPS and AP-3 complexes. We find that as many as five members have been observed to affect the CG pathway-these include Lightoid, Carmine and White, in addition to Dor and Car. That these genes interact genetically with one another [70] suggests that they may regulate the capacity of the CG pathway. This is reminiscent of the master transcriptional regulator of lysosomal biogenesis, which acts through CLEAR elements [71]. It follows that we see seven genes (not shown) containing CLEAR elements that play a role in the CG pathway, whereas only one such gene plays a role in the CD pathway. This may indicate a preference for the CG pathway in crosstalk between endocytic routes and the dynamic regulation of lysosomal biogenesis.

The discovery of a role for V1 subunit of the V-ATPase in the CG pathway coupled with the observation of rapid onset of acidification of GEECs [11], indicates a central role for pH regulation in the CG uptake mechanism. An interesting prospect here is that acidification of GEECs regulates the return of key players important for CG endocytosis, and provides a mechanism to couple the huge flux of recycling membrane to internalization at the cell surface. This mechanism may play a role in regulating membrane homeostasis, and uncovering the molecular machinery behind this is an important goal for future experimentation.

### Conclusions and prospects

We have screened for core and peripheral molecular players underlying endocytosis. An internal statistical analysis of the primary assay establishes a 10% false-positive rate (though about 30% of the genes tested for a given feature do slip through the net). The high rate of feature-specific recall in the classification assay, as well as the correlation of the direction of endocytic perturbation in the secondary assay compared to those observed in mutant cell lines, provides further confidence in our methodology. Our hierarchical classification gives us a sense of the peripheral genes that control CD and CG endocytosis. Deeper analysis reveals a set of new core regulators of clathrin-independent endocytic processes in metazoa: the components hVPS33a and SYX1, as well as the regulatory control of the tension sensing mechanism and vacuolar acidification. Apart from these specific results, the set of PVs for all the genes screened represents an information-rich resource that can be used to test hypotheses beyond those we have considered here. We have placed this resource online (http://rnai.ncbs.res.in/endosite) and hope it will stimulate further study into the molecular basis of endocytic routes and intracellular transport.

## Materials and Methods

### Cell culture, Fly stocks, RNAi and stable lines

*Drosophila* S2R+ cells were grown and treated for RNAi as before [8]. dsRNA was prepared from the *Drosophila* Open Biosystems library v1 (Table S9; [60], [72], [73] as described [8]. dsRNA against bacterially encoded zeocin and mock transfections were used as negative controls. For seeding onto cell arrays, 10 ng of dsRNA was dried onto the cell array added to a final volume of 2 µl of Schneider's *Drosophila* Insect Medium (SDM; Invitrogen) containing ∼100–150 cells. Cell arrays were incubated in a humid chamber for 72 hours before processing. Chinese Hamster Ovary (CHO) cells stably expressing FR-GPI and human TfR (IA2.2 cells) or Human AGS cells (ATCC catalog No:CRL-1739) and FR-AGS (Human Folate receptor expressing AGS cells) were used for mammalian endocytic assays. They were grown in HF-12 or RPM1 media (CHO,AGS/FR-AGS respectively; HiMEDIA, Mumbai, India) containing NaHCO3 and penicillin, streptomycin (100 µg/ml) and supplemented with 10% FBS (GibcoBRL, Rockville, MD). Mouse embryonic fibroblasts (MEFs) were grown and treated as described previously [9], [13].

### Endocytic uptake experiments and perturbations

For CD and CG uptake experiments, S2R+ cell arrays were incubated with endocytic probes Fdex and Alexa568-Tf in M1 buffer (150 mM NaCl, 5 mM KCl, 1 mM CaCl2, 1 mM MgCl2, 20 mM HEPES, pH 6.9) supplemented with BSA (1.5 mg/ml) and D-glucose (2 mg/ml) at room temperature (21–24°C) for 20 minutes, and extensively washed in the same medium (see Figure **1A** for schematic outline and Supplementary Information for details on probes and endocytic assays). In the case of genes that affect TfR uptake it is necessary to monitor external levels of TfR along with internal levels in each cell, as quantitative uptake is revealed by internal∶external ratios of Tf. To quantitate the specific endocytic uptake of TfR, cells were incubated with fluorescent conjugates of Tf (along with fluorescent conjugated 10 kDa dextran (Molecular Probes, OH)) at room temperature for different pulse times and then washed in M1 and placed on ice. Ascorbate buffer with 50 µg/ml desferroxamine mesylate (pH 4.5; 10 min on ice) treatment was carried out to remove cell-surface Tf. Surface pools of receptors were labeled by further incubation on ice with antibodies to anti-hTfR (αOkt9) for 30 min. Endosomal pH was neutralized by the addition of ammonium chloride (30 mM in M1) post-fixation so as to dequench pH-sensitive fluorochromes. Subsequently, cell arrays were washed, fixed (2.5% paraformaldehyde in M1), mounted in Vectashield (Vector Labs, CA) and imaged at 20× 0.75 NA with an automated microscope with manually guided focusing [8]. For pulse-chase classification assays, either Alexa 568-Tf/Fdex or Fdex alone was pulsed for 5 minutes, and the Fdex only population was followed by a chase of 12 minutes and a second pulse of another fluid-phase probe (TMR-Dex) for an additional 3 minutes (Figure **3A**).

CHO cells were treated with Bafilomycin (250 nM), Brefeldin A (20 µg/ml) or Monensin (50 µM) (Sigma-Aldrich) and CG endocytosis was monitored using 1 mg/ml TMR-dextran pulse of 10 minutes. The drugs were maintained throughout the pulse and the uptake was terminated using chilled M1. In the combined assay, the drugs were treated sequentially with pulse of TMR-Dextran containing the drugs during the assay. Details of time points with inhibitors is provided in Supplementary Information.

For mammalian RNAi, sub-confluent cells (50–60%) were transfected with the corresponding shRNA -Syntaxin1A-eGFP shRNA (vector control, pG-SUPER empty vector) using Fugene 6 transfection reagent according to supplied protocol. For siRNA treatments, cells were transfected with 150 ngs siRNA using HiPerFect transfection reagent (Qiagen) for 60 hours according to the recommended protocol. Human Syntaxin1A, Human VPS33A and Human VPS33B ONTARGET plus siRNA SMARTpool were acquired from Dharmacon RNAi Techniologies (Thermo Fisher Scientific). RNAi-treated cells were assayed for CG or CD-endocytosis 60–72 hours post transfection as described above. Anti-CD44 and Tf-555 or horseradish peroxidase (HRP) endocytic assays in MEFs, followed by electron or fluorescence microscopy were all exactly as described [9], [13].

### Primary cell cultures

Flies that were mutant for a few genes (see Table **S5**), identified as affected (or not) in CG endocytosis in the characterization assay were obtained from the Bloomington Stock Centre at Indiana University. CG endocytosis was examined in primary hemocytes derived from wild type or mutant animals as described [7]. Many of the alleles (indicated by blue letters in **ST5**) were not viable as adults. These were obtained as heterozygous mutants over appropriate chromosomal balancers (FM7, CyO or TM6, for the 1st, 2nd or 3rd chromosome, respectively). Since these markers can only be identified in adult stage, they were crossed to flies containing GFP-tagged balancers. GFP –expressing adult males and females carrying the mutant allele in heterozygous condition, were selected from this cross and allowed to mate. From this cross, larvae that were non–green (ie. homozygous for mutant allele) were used for the assays. Primary hemocytes from wild type and mutant larvae were pulsed with the two fluid probes (as done in the characterization assay) and fixed and imaged at high resolution (60×, 1.4 NA). Assays were repeated twice or more for each allele; alleles for which no-non green larvae were obtained were likely lethal before the 3rd instar larvae (orange letters in Table **S5**).

### Imaging and image processing

Quantitative imaging and image processing of mammalian cells and *Drosophila* hemocytes has been described previously [7], [9], [11], [13]. Analysis of image-based features for S2R+ cells is described in detail in Table **S1** and summarized here. We analyzed 27 different image-based parameters: Fint1–3/Tint1–3: Average per cell intensity in Fdex or TfR channel with different background subtractions, respectively; Fint4/Tint4: Fraction of cell with non-zero Fdex or TfR signal; Fmph1/Tmph1: average size of Fdex or TfR endosome; Fmph2/Tmph2: Fraction of cell area occupied by endosomes; Fmph3/Tmph3: average circularity ratio of endosomes; Fnum/Tnum: average number of endosomes; Okt: average intensity of the surface levels of TfR as marked by αOkt9; Rto1–3: only for the TfR channel, represents the surface level normalized pool of internal TfR ie. Tint1/Okt or Tint2/Okt or Tint3/Okt respectively; Fclc/Tclc: the fraction of Fdex endosomes that colocalize with TfR endosomes, and vice versa; NucSize/NucCirc/NucFluct/NucDist/CellSize: measures of nuclear and cell size.

### A Z-score to quantify shape changes of phenotypic distributions

To quantitatively distinguish between distributions, we use the test statistic from the Kolmogorov-Smirnov test for two distributions. This test is non-parametric, which means that it makes no assumptions about the actual shapes of the distributions being tested. The Kolmogorov-Smirnov test statistic [28] is defined as the maximum vertical deviation between two cumulative distribution functions. Our Z-score is a scaled version of this statistic based on pooled comparisons between test wells and negative control wells on every slide (See Supplementary Information).

### Tree construction

Algorithms used for phylogenetic analysis can generally be used to hierarchically cluster any type of data. Here we use a parsimony approach to build our tree. We regard the 27 features as “species”, and the 7131 profiled genes as binary “characters”. If a certain gene is observed to influence a certain feature, that entry of the 27×7131 matrix is set to ‘1’; otherwise, it is set to ‘0; that is, each column of this matrix is the 27-dimensional feature vector. The tree describes the observed distribution of characters at each leaf (each feature) with the minimal number of character (gene) gain and loss events on internal nodes. This structure can be judged a good description of the data if (1) we observe only character gains, so a gene added at an ancestral node occurs in all descendants of that node; and (2) if each character is associated with a single gain event, so that a gene which influences two nodes also influences all others descended from their common ancestor. In practice we observe only a small number of character losses (280 out of 1881 total events), which we interpret as due to the false-negative rate of our assay; we therefore focus only on the genes added at each node. To quantify multiple gain events, we examined the overlap between genes added at pairs of nodes, and tested this against the null hypothesis that they are selected at random from the background of hits. Node pairs were significantly depleted in common genes, with a single interesting exception - Fdex and Tf colocalization (which are related by definition). Once this overlap is removed, only 203 out of 1072 characters show multiple gains (Figure **2C**). Housekeeping genes such as RNA polymerase subunits, which are close to the root of the tree and affect almost all features, do not provide much insight into the mechanisms of endocytosis. In contrast, genes which influence a restricted set of processes without affecting any others are interesting targets for future study. Maximum parsimony trees were constructed using PHYLIP [74]. We first restricted the set of characters to 505 non-trivial genes, those which were hits for two or more features. We used ‘seqboot’ to generate 100 datasets using the half-jackknife option. For each dataset we used ‘pars’ to construct maximum parsimony trees, in each case selecting the best tree from 25 runs with shuffled species order. We used ‘consense’ with the majority rule option to generate a consensus tree (Figure **S5**). We then re-ran ‘pars’ on the consensus tree and stored the inferred set of characters at each internal node. Finally, assuming the least populated internal node to be the root, we determined the set of genes added at each node.

### GO-guided tree construction

In this procedure, the leaves of the individual features are now populated with GO cellular component annotations instead of their CG numbers. The parsimony algorithm shifts shared GO annotations to higher nodes in the tree; generic annotations (cytoplasm, nucleus, membrane, intracellular, extracellular region, plasma membrane) converge at the root of the tree.

### Network prediction

Genes belonging to a given secondary quadrant were tested for functional association with GeneMania [75]. We used three main criteria for association (genetic, physical and predicted interactions) and selectively omitted co-expression and co-localization so as to rule out less specific networks. We selected the GO biological process based association option and added up to 20–50 additional genes into the predicted networks to enhance searching. With these conditions, approximately 50–80% of genes in the quadrants highlighted (Figure S**6**) were captured in networks. We considered functional GO annotations as enriched at p values<10^−3^ within the GeneMania network report.

### Enrichment calculations

#### Orthologs and screens enrichment

The standard procedure for determining the annotation enrichment of a list (orthologs/screen hits) within a given node, consists of calculating the probability of drawing the same or higher number of study set genes annotated to the term if we selected a list of genes of the same size as the study set randomly from the population set. Enriched nodes were the ones which had a P value of 0.05 or lesser post multiple hypothesis correction.

#### Interactions enrichment

The number of interactions present at each node of the tree was calculated for the real dataset. 10000 permuted interaction datasets were constructed, keeping the total number of interactions constant. For each permutation, the total number of interactions present in each node was calculated. The probability of drawing the same or higher number of interactions within each of these nodes for the randomized trials was calculated as an empirical p-value. Nodes with a p-value of 0.05 or less (post-multiple hypothesis correction) were considered statistically enriched for interactions.

#### String database parameters

Protein-protein interaction data for *D. melanogaster* was obtained from the STRING v8.3 database (www.string-db.org) using a confidence score filter of > = 0.400. The prediction method was set to ‘All methods’, which represents a combination of 7 different prediction methods used by STRING to incorporate protein-protein interactions (Jensen et al., 2009).

## Supporting Information

Figure S1 **Primary Screen and post processing.** (A) The ‘cell array’ platform used for the screen is a clear borosilicate glass slide (25×75 mm) printed with hydrophobic ink (Erie Scientific, OR) to create 300 wells (30×10) of 1 mm diameter each, with a working volume of 2 µl/well and an inter-well spacing of 1 mm. The hydrophobic mask prevents mixing of soluble content between wells while allowing assays based on multiple liquid exchange steps to be performed easily and with high time resolution without the use of robotics. Pertains to Figure 1A. (B,C) Three randomly selected unprocessed images from three different slides, acquired at 16 bit depth in the Fdex (B) or Tf (C) channels, and displayed here without any rescaling or background subtraction. Graphs on the right show histograms of the pooled raw pixel intensities from all the images in the relevant channel from a single slide (1500*512*512 pixels). During acquisition of all images in the screen, care was taken to ensure that the maximum intensity in each image is far below saturation (<20000 of 65536 or 2∧16 possible grayscale levels). (D) Sample Fdex image highlighting the image processing steps used to extract information at different spatial scales. Each image was subjected to semi-local or local (tophat) background subtraction using a morphological disk of varying size (large: 64 pixel radius; medium: 10 pixel radius; small: 5 pixel radius). The large disk was chosen to exceed the size of the largest possible cell, ensuring the subtraction of the global background, while the medium and small disks were chosen to emphasize different aspects of the endosome distribution (large: total cellular fluorescence, medium: large endosomes and un-resolved clumps, small: individual bright and dim endosomes). Per-cell information from these three processed images corresponds to the parameters Fint1, Fint2 and Fint3 respectively. The same transformation was applied to the Tf images with slightly different disk sizes (large: 64; medium: 14; small: 7; to account for the tubular morphology of Tf endosomes). (E) The prevalence of metagenes (left, genes conserved across multiple phyla [21]) and genes with dsRNAs predicted to have multiple off-target effects (right) in the set of hits (green) relative to the entire library (black). The data indicate a slight enrichment for metagenes in the hit-set. We identified dsRNAs with at least one potential off-target ([77], DRSC, http://www.flyrnai.org/RNAi_find_frag_free.html), and found no enrichment within the set of hits (bars on the right), pertains to the properties of genes detailed in Table S1. (F) Plot quantifying the fraction of hits determined to be detectably expressed (green) or absent (black) by transcriptome analysis of S2R+ cells [78] across multiple screens carried out in this cell line. The data shown here include screens for N-FAT activation (NFAT) [79], Wingless signaling (Wingless) [80], store operated Ca^+2^ entry (Cracm1) [81], light-dependent CRY degradation (CRY) [82] and nuclear import of SMADS (Msk) [83] in addition to this screen. Pertains to the properties of genes detailed in Table S1.
(PDF)

Figure S2 **Negative control distributions.** (A) Cumulative distributions (cdfs) of single cell intensity distributions from 30 negative control wells, shown here after affine-normalization (by subtracting the mean and dividing by the standard deviation). Each graph shows the negative controls from a different slide. (B) Frequency histogram of Z-scores, with positives (panel 1–4), unused negatives (panel 5) and test genes (panel 6) plotted separately. Potential hits are picked at a threshold> = 3. The final selection of a hit depends on how a gene performs in triplicate.
(PDF)

Figure S3 **Sec23 affects fluid-phase uptake in mammalian cells.** (A, B) Human AGS (wild type) cells were transfected with SEC23A shRNA (targeting bases 494–512 in human SEC23A mRNA (accession #NM_006364)) encoded in pG-Super vector or empty pG-Super vector (control) in separate dishes, and transfected cells were identified by EGFP expression ∼60 h after transfection. Endocytosis via the CLIC/GEEC pathway was assessed by TMR-Dex uptake (A) and CD-endocytosis was monitored by uptake of Cy5-labelled transferrin (Cy5-Tf) normalized to the surface level expression of transferrin receptor as ascertained by measuring the amount of Cy3-labelled Okt9 antibody (Cy3-αOkt9) against the transferrin receptor (B). For these experiments, cells were pulsed for 5 min at 37°C with TMR-Dex (1 mg/ml) or Cy5-labelled transferrin (Cy5-Tf) and fixed and imaged at 20×. Micrographs of representative fields of cells from brightfield (BF) EGFP and the respective endocytic (TMR-Dex and Cy5-Tf) and surface (Cy3-αOkt9) are shown in the panels on the left. In (A), a histogram from one representative experiment shows the measured integrated intensities of TMR-Dex uptake per cell in each condition; the error bars here represent the weighted mean of fluorescence intensities ± SEM (n>50 cells per replicate, 2 replicates per experiment). In the upper panel in (B), the histogram shows the integrated intensities of internalized Cy5-Tf in SEC23A shRNA transfected or control cells (normalized to control values; white bars), and the corresponding surface level expression of TfR as measured by Okt9 binding also normalized to control values (black bars). The error bars represent the weighted mean of fluorescence intensities ± SEM (n>40 cells per replicate, 2 replicates per experiment). The second histogram represents uptake of Tf (normalized to surface TfR expression level) in the same transfected cells plotted as an internal∶external ratio for each condition (Lower panel). (**C**) AGS cells were transfected with SEC23A shRNA or control vector in separate 90 mm dishes. Transfected cells from each dish were separated by FACS on the basis of the EGFP expression at two different time points, 48 hours post transfection and 72 hours post transfection. Lysate from each of these time points were used to carry out a Western blot. In the right panel, the amount of SEC23A protein as detected by anti-SEC23A (kind gift of Benjamin Glick, U. Chicago) on western blots is quantitated and normalized to the actin level (as a loading control) per lane. Bars, 20 µm.
(PDF)

Figure S4 **Enrichment of protein-protein interactions within nodes of the tree.** (A) Based on the distribution of genes in the nodes and leaves of the tree (Figure 3; Table S2) intra-node protein-protein interactions were identified using parameters collected from STRING. The total number of interactions present in each node was calculated and the probability of drawing the same or higher number of interactions across 10000 randomized trials was estimated. Nodes with a *p*-value≤0.05 (post-multiple hypothesis correction) were considered to be enriched for interactions, represented as pixels in the heatmap. A subset of nodes is significantly enriched with respect to orthologs in other species, potentially indicating the presence of evolutionarily conserved hubs. (B) Interaction maps of nodes enriched for interactions in *Drosophila*. Genes are color-coded based on the presence of orthologs in yeast (yellow), humans (blue), or both yeast and humans (green).
(PDF)

Figure S5 **Bootstrap results for the primary tree.** 100 replicate trees were generated by a half-jackknife operation, leaving out half the genes at random for each replicate. The final tree is a consensus of these 100 replicates. The bootstrap support for each branch (the fraction of replicates in which this branch occurs) is shown.
(PDF)

Figure S6 **Highlighted networks involved in protein degradation, the stress response, cell proliferation and the organization of the cytoskeleton.** (A) Genes that affect both CG and CD pathways negatively (intersecting quadrants from Figure 5; see main text) were overlaid on a protein-protein interaction map (GeneMania; [84], with selected GO annotations highlighted in different colors (see legend). (B) Genes involved in cytoskeleton organization were selected using GO annotations, and were assayed for their roles in the CG pathway using the assay detailed in Figure 4. Complete scores are listed in Table S6. A network analysis [84] on the pooled set of results from this additional assay and the screen (Tables S1 and S4) highlights the significance of specific cytoskeletal elements in the two pathways of interest. The color of each edge in the network denotes the type of interaction (see legend below) and the fill color for each node denotes the functional category (see legend above network). Classification of the gene into a CG pathway hit, CD pathway hit, or a common hit is denoted by the letters F, T, or FT.
(PDF)

Figure S7 **CG and CD pathways assessed in primary larval hemocytes from** ***drp*** **and** ***Flo-1*** **mutants.** (A,B) Fluorescent dextran (fluid) and anionic ligand binding receptor uptake of Cy3-malelylated BSA (receptor) in *drp1* (DRP) and *Flo-1* (FLOT) mutant hemocytes as compared to those from wild type CS flies. Graphs represent means ± SEM of relative fluorescence intensities (RFU) from 3 independent experiments (n = 10–20 cells per experiment). In each case, differences were not statistically significant from controls (Student T-test, *p*>0.05). (C) Representative micrographs of CS or mutant hemocytes (*dor^4^*, *car^1^*) that were pulsed for 3 min with fluorescent dextran and then chased for 12 min. As shown previously [47], these late endosomal mutants fail to traffic cargo to lysosomes and thus accumulate probe during the chase. See Figure 5B for quantifications.
(TKF)

Figure S8 **Effect of depletion of Car (hVps33) and Syntaxin 1 on levels of Syntaxin 1 and CD endocytosis.** (A, B) Histograms show uptake of Tf (normalized to cell surface TfR (Okt9) staining levels) in Car-depleted S2R+ cells (A) and uptake of Cy3-malelylated BSA (receptor) in car1 mutant hemocytes (B). Note that there appears to be no significant difference in the uptake of bona fide CD-cargo in both cases when compared to control cells. Inset in A shows the protein levels of Car in the dsRNA-treated cells compared to untreated cells by western blot. Actin staining (at 40 kD) from the same western blots was used as a loading control. (C) Histogram shows that normalized Tf uptake in Syx1A-depleted S2R+ cells was no different from that measured in control cells as seen in a single representative experiment out of three independent experiments (n>50 cells per replicate, 2 replicates per experiment). (D) Levels of Syx1A were measurably different in Syx1-depleted cells (immunofluorescence, n>50 cells per replicate from 2 replicates). (E) The amount of Syx1A is reduced in Car-depleted cells. Graph represents normalized data from a representative experiment with n>50 cells per treatment from 2 replicates. (F) Histogram shows that normalized Tf uptake is unaffected in human AGS cells depleted of SYX1A, VPS33A and VPS33B.
(PDF)

Table S1 **Z-scores for the entire screen, the hits and parameter descriptions.** Primary Z-Scores (tab labeled “Z-score”) calculated for the 7216 genes tested in triplicate across all 27 parameters (details in tab labeled “Parameter Description”), and the corresponding parameter-wise list of hits (tab labeled “Hits-Parameter wise”) obtained using a KS-based Z-Score with a threshold of Z> = 3 for at least two out of three replicates.
(XLS)

Table S2 **Distribution of hits within the nodes and leaves of the parsimony-generated tree structure.** Distribution of hits within the nodes and leaves of the parsimony-generated tree structure described in Experimental Procedures and Figure 2. In addition, hits annotated to be membrane-associated, involved in signal transduction, or specifically annotated to be kinases (by Gene Ontology) are detailed separately in additional tabs to highlight functional categories of potential interest.
(XLS)

Table S3 **Tree classification of GO terms associated with the set of hits.** Distribution of Gene Ontology Cellular Component (GO_CC) annotations within the nodes and leaves of the parsimony-generated tree structure, utilizing GO_CC guided pull-up methods (see Experimental Procedures and Figure **2**).
(XLS)

Table S4 **Detailed results from the high-resolution classification assay.** *p* values and normalized means (relative to the local control wells, as described in Experimental Procedures) for genes tested in the classification assay for CG or CD pathway activity as indicated. Additional tabs represent quadrant-wise (Figure **4**) distribution of genes at a *p* value<0.10.
(XLS)

Table S5 ***Drosophila*** **strains used in this study.** Detailed description of the *Drosophila* mutant lines screened for their effects on the CLIC/GEEC pathway using primary hemocytes from viable and wandering third instar larvae.
(XLS)

Table S6 **Gene lists from other genome-wide RNAi screens.** Tabs contain lists of hits overlapping between this screen and multiple other siRNA screens grouped into functional categories.
(XLS)

Table S7 **Cytoskeleton-related genes tested in the high-resolution classification assay.** *p* values (Student's T-test) and normalized means of a sub-set of cytoskeletal genes selected based on their Cytoskeleton GO classification. Genes not tested in the primary screen are highlighted in red. This subset of genes was tested separately in the high-resolution classification assay (Figure **S6**), to assess their roles in the CG pathway.
(XLS)

Table S8 **Comparison with other screens for endocytosis.** List of genes already classified as endocytosis genes by KEGG (Endocytosis genes) and the hit lists from other recent endocytosis or lipid droplet screens (as indicated) compared to the set of hits from this screen.
(XLS)

Table S9 **Sequences of dsRNA primers used in the primary screen.**
(XLS)

Methods S1 (DOCX)
