# Correction: Population Distribution Analyses Reveal a Hierarchy of Molecular Players Underlying Parallel Endocytic Pathways

**DOI:** 10.1371/journal.pone.0204770

**Published:** 2018-09-21

**Authors:** Gagan D. Gupta, Gautam Dey, Swetha MG, Balaji Ramalingam, Khader Shameer, Joseph Jose Thottacherry, Joseph Mathew Kalappurakkal, Mark T. Howes, Ruma Chandran, Anupam Das, Sindhu Menon, Robert G. Parton, R. Sowdhamini, Mukund Thattai, Satyajit Mayor

In addition to the noted affiliation, Ruma Chandran is affiliated with: School of Life Sciences, Manipal Academy of Higher Education, Manipal, Karnataka, India.
