# Supplementary figures and images for: Population Distribution Analyses Reveal a Hierarchy of Molecular Players Underlying Parallel Endocytic Pathways
Source: PLoS One. 2014 Jun 27;9(6):e100554. doi: 10.1371/journal.pone.0100554 (PMC4074053; doi:10.1371/journal.pone.0100554)

A

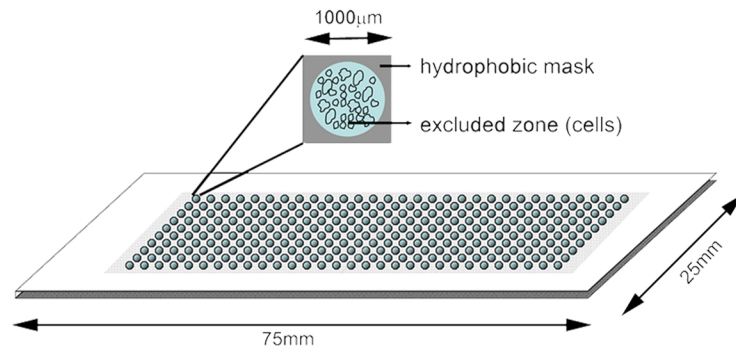

B

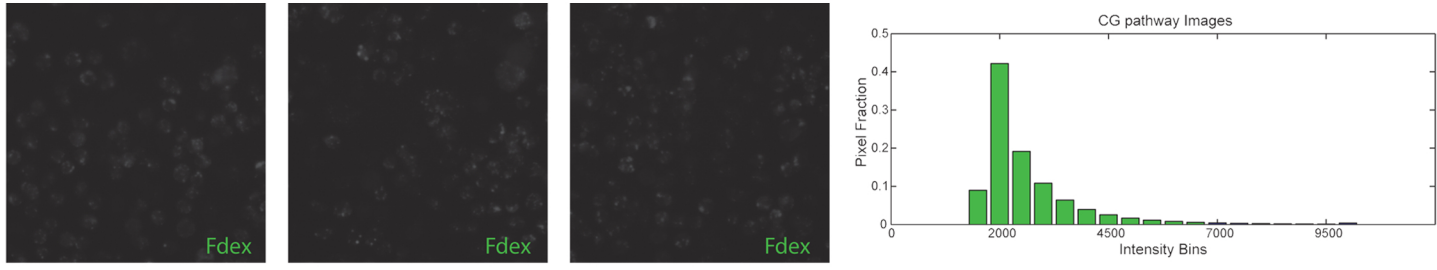

C

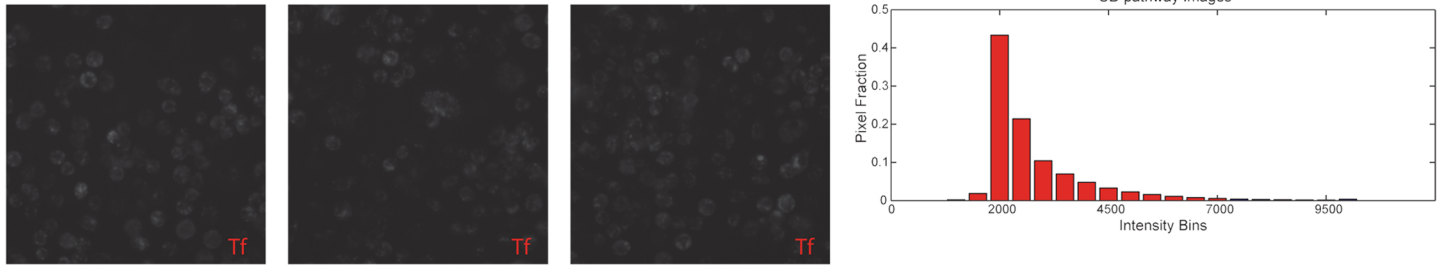

D

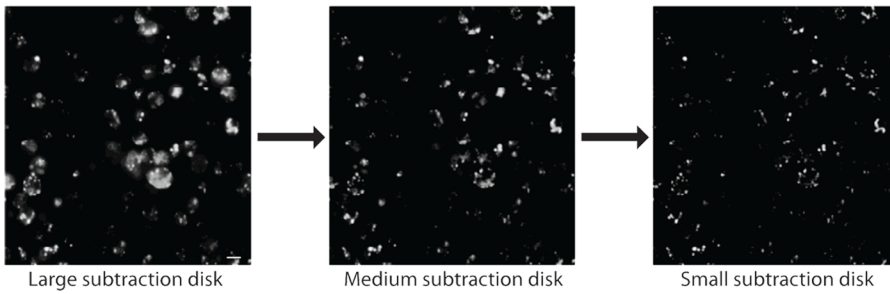

E

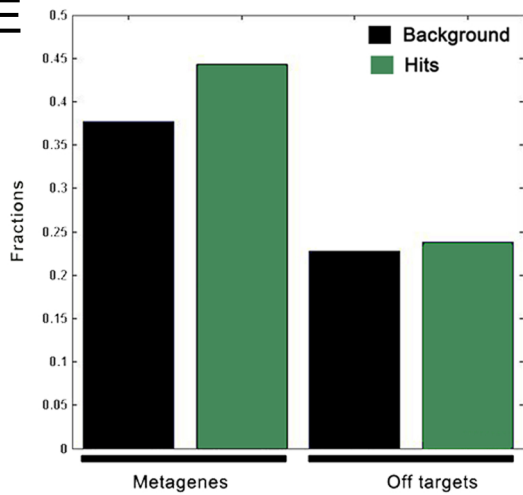

F

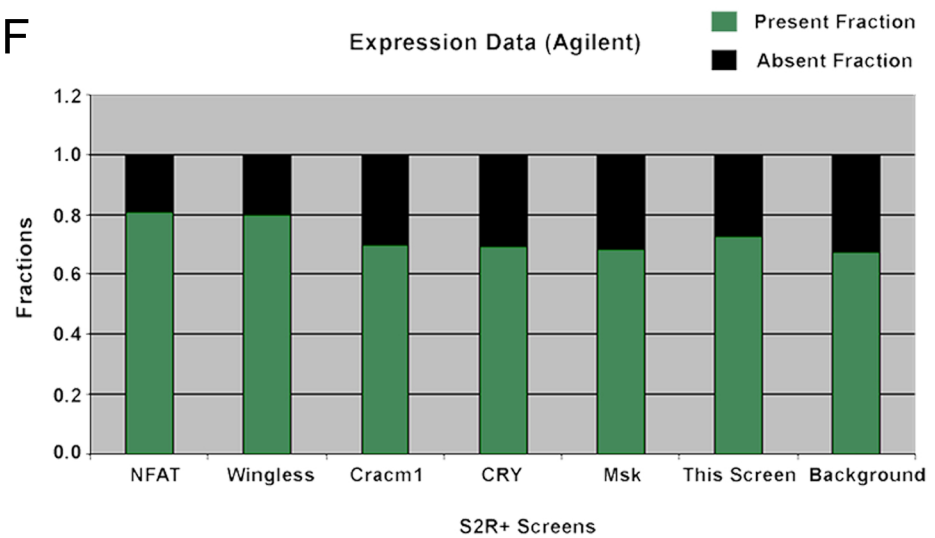

Supplement: Figure S1 — Primary Screen and post processing. (A) The ‘cell array’ platform used for the screen is a clear borosilicate glass slide (25×75 mm) printed with hydrophobic ink (Erie Scientific, OR) to create 300 wells (30×10) of 1 mm diameter each, with a working volume of 2 µl/well and an inter-well spacing of 1 mm. The hydrophobic mask prevents mixing of soluble content between wells while allowing assays based on multiple liquid exchange steps to be performed easily and with high time resolution without the use of robotics. Pertains to Figure 1A. (B,C) Three randomly selected unprocessed images from three different slides, acquired at 16 bit depth in the Fdex (B) or Tf (C) channels, and displayed here without any rescaling or background subtraction. Graphs on the right show histograms of the pooled raw pixel intensities from all the images in the relevant channel from a single slide (1500*512*512 pixels). During acquisition of all images in the screen, care was taken to ensure that the maximum intensity in each image is far below saturation (<20000 of 65536 or 2∧16 possible grayscale levels). (D) Sample Fdex image highlighting the image processing steps used to extract information at different spatial scales. Each image was subjected to semi-local or local (tophat) background subtraction using a morphological disk of varying size (large: 64 pixel radius; medium: 10 pixel radius; small: 5 pixel radius). The large disk was chosen to exceed the size of the largest possible cell, ensuring the subtraction of the global background, while the medium and small disks were chosen to emphasize different aspects of the endosome distribution (large: total cellular fluorescence, medium: large endosomes and un-resolved clumps, small: individual bright and dim endosomes). Per-cell information from these three processed images corresponds to the parameters Fint1, Fint2 and Fint3 respectively. The same transformation was applied to the Tf images with slightly different disk sizes (large: 64; me [file pone.0100554.s001.pdf]

A

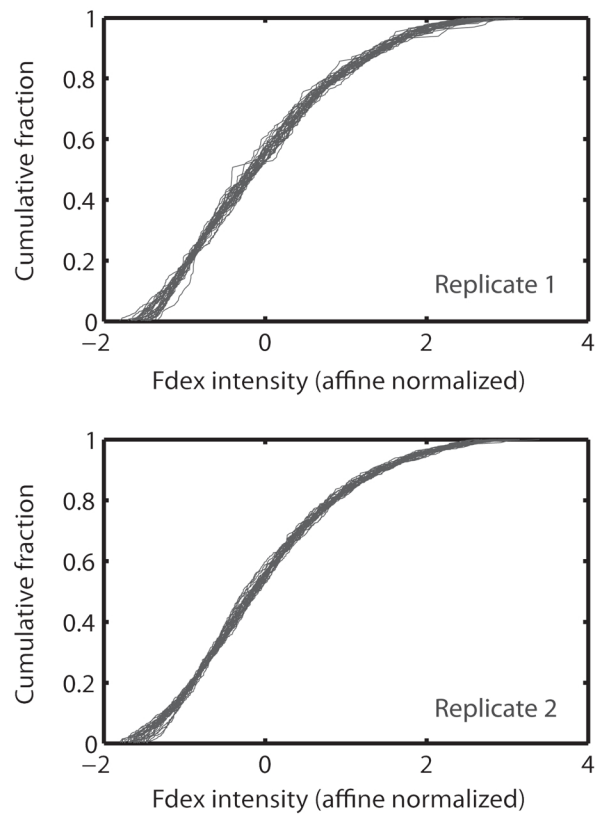

B

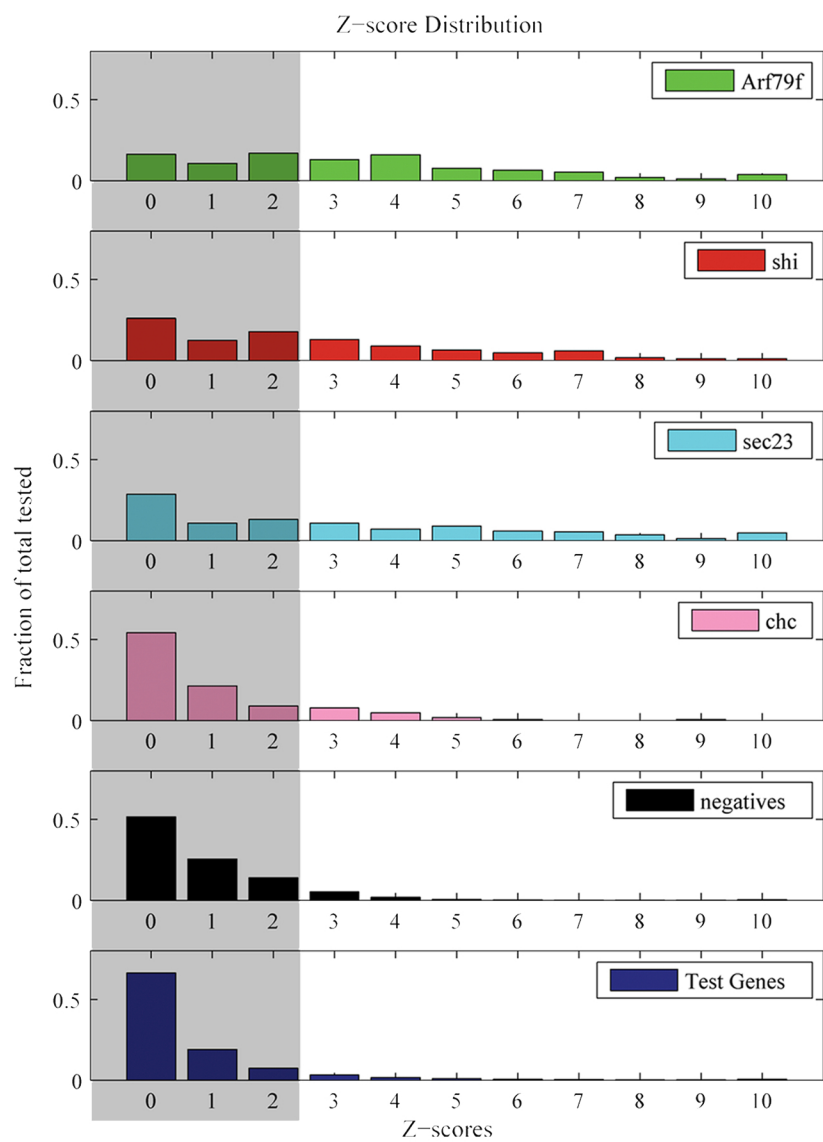

Supplement: Figure S2 — Negative control distributions. (A) Cumulative distributions (cdfs) of single cell intensity distributions from 30 negative control wells, shown here after affine-normalization (by subtracting the mean and dividing by the standard deviation). Each graph shows the negative controls from a different slide. (B) Frequency histogram of Z-scores, with positives (panel 1–4), unused negatives (panel 5) and test genes (panel 6) plotted separately. Potential hits are picked at a threshold> = 3. The final selection of a hit depends on how a gene performs in triplicate. (PDF) [file pone.0100554.s002.pdf]

**A**

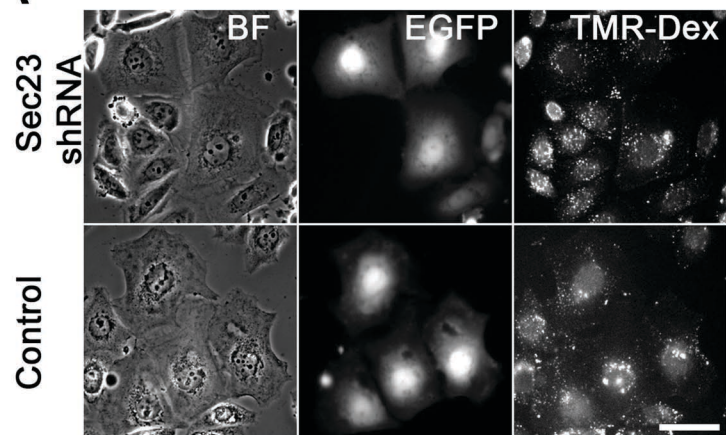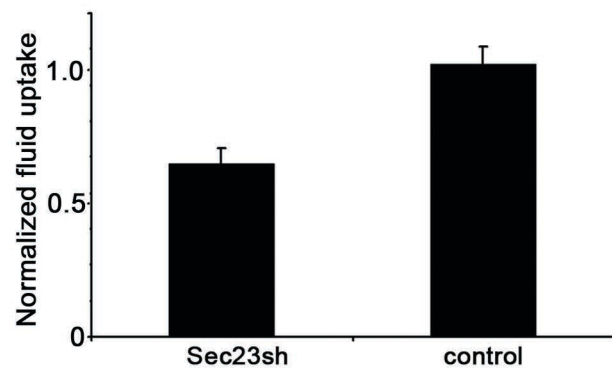

**B**

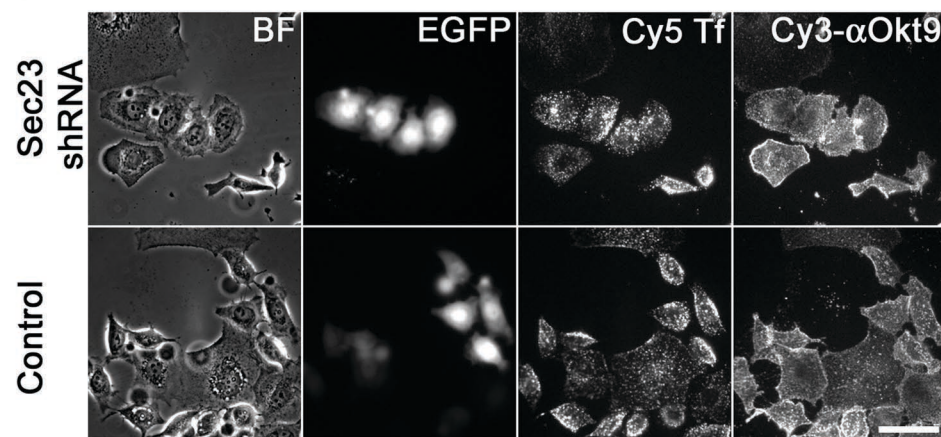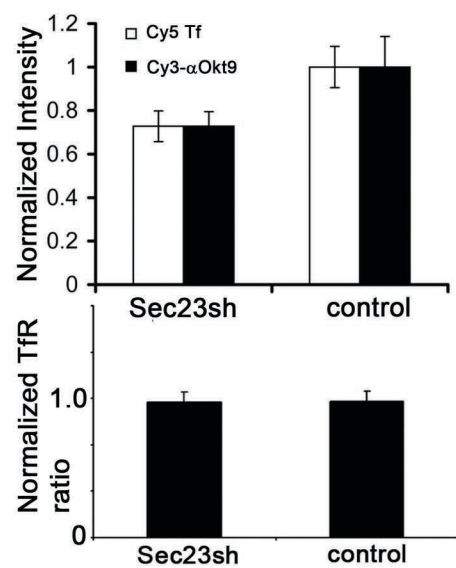

**C**

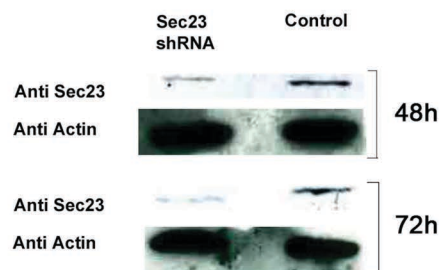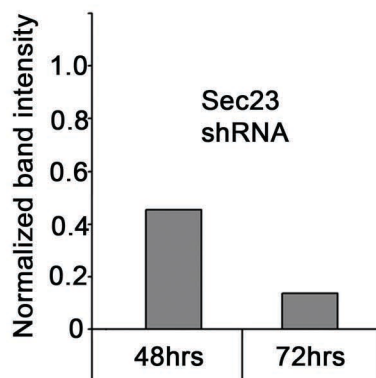

Supplement: Figure S3 — Sec23 affects fluid-phase uptake in mammalian cells. (A, B) Human AGS (wild type) cells were transfected with SEC23A shRNA (targeting bases 494–512 in human SEC23A mRNA (accession #NM_006364)) encoded in pG-Super vector or empty pG-Super vector (control) in separate dishes, and transfected cells were identified by EGFP expression ∼60 h after transfection. Endocytosis via the CLIC/GEEC pathway was assessed by TMR-Dex uptake (A) and CD-endocytosis was monitored by uptake of Cy5-labelled transferrin (Cy5-Tf) normalized to the surface level expression of transferrin receptor as ascertained by measuring the amount of Cy3-labelled Okt9 antibody (Cy3-αOkt9) against the transferrin receptor (B). For these experiments, cells were pulsed for 5 min at 37°C with TMR-Dex (1 mg/ml) or Cy5-labelled transferrin (Cy5-Tf) and fixed and imaged at 20×. Micrographs of representative fields of cells from brightfield (BF) EGFP and the respective endocytic (TMR-Dex and Cy5-Tf) and surface (Cy3-αOkt9) are shown in the panels on the left. In (A), a histogram from one representative experiment shows the measured integrated intensities of TMR-Dex uptake per cell in each condition; the error bars here represent the weighted mean of fluorescence intensities ± SEM (n>50 cells per replicate, 2 replicates per experiment). In the upper panel in (B), the histogram shows the integrated intensities of internalized Cy5-Tf in SEC23A shRNA transfected or control cells (normalized to control values; white bars), and the corresponding surface level expression of TfR as measured by Okt9 binding also normalized to control values (black bars). The error bars represent the weighted mean of fluorescence intensities ± SEM (n>40 cells per replicate, 2 replicates per experiment). The second histogram represents uptake of Tf (normalized to surface TfR expression level) in the same transfected cells plotted as an internal∶external ratio for each condition (Lower panel). (C) AGS cells were transfected with SEC23A shRN [file pone.0100554.s003.pdf]

A

## Interactions Enrichment

Tree Categories

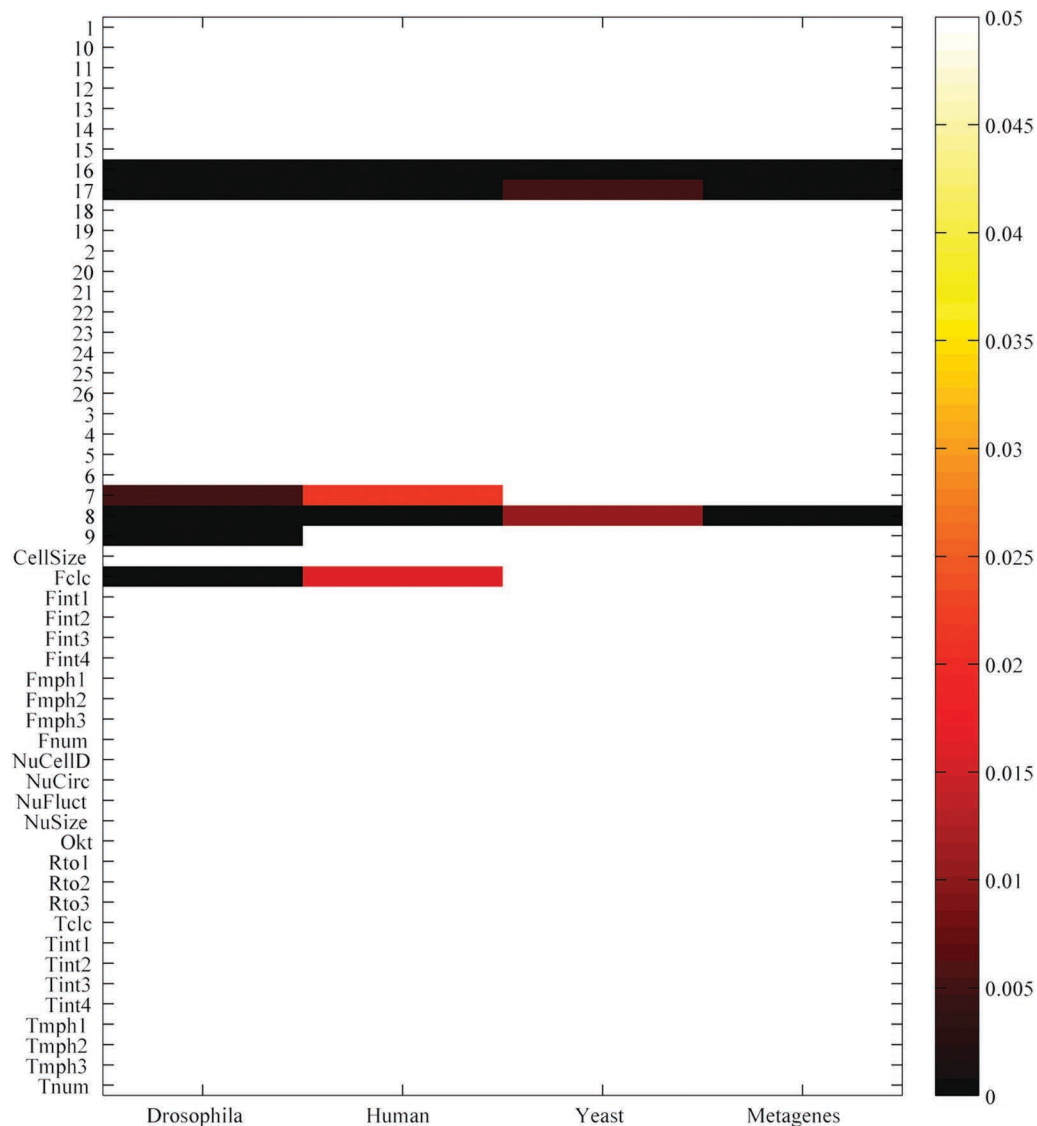

B

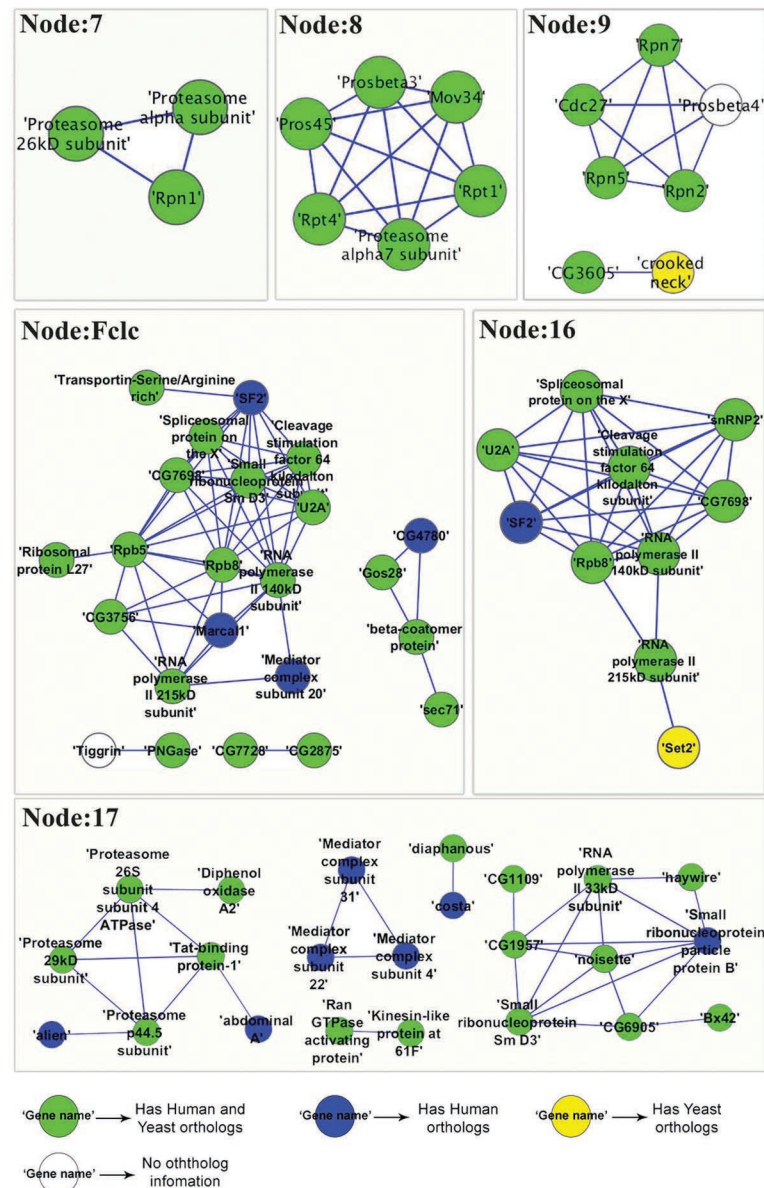

Ortholog Categories

Supplement: Figure S4 — Enrichment of protein-protein interactions within nodes of the tree. (A) Based on the distribution of genes in the nodes and leaves of the tree (Figure 3; Table S2) intra-node protein-protein interactions were identified using parameters collected from STRING. The total number of interactions present in each node was calculated and the probability of drawing the same or higher number of interactions across 10000 randomized trials was estimated. Nodes with a p-value≤0.05 (post-multiple hypothesis correction) were considered to be enriched for interactions, represented as pixels in the heatmap. A subset of nodes is significantly enriched with respect to orthologs in other species, potentially indicating the presence of evolutionarily conserved hubs. (B) Interaction maps of nodes enriched for interactions in Drosophila. Genes are color-coded based on the presence of orthologs in yeast (yellow), humans (blue), or both yeast and humans (green). (PDF) [file pone.0100554.s004.pdf]

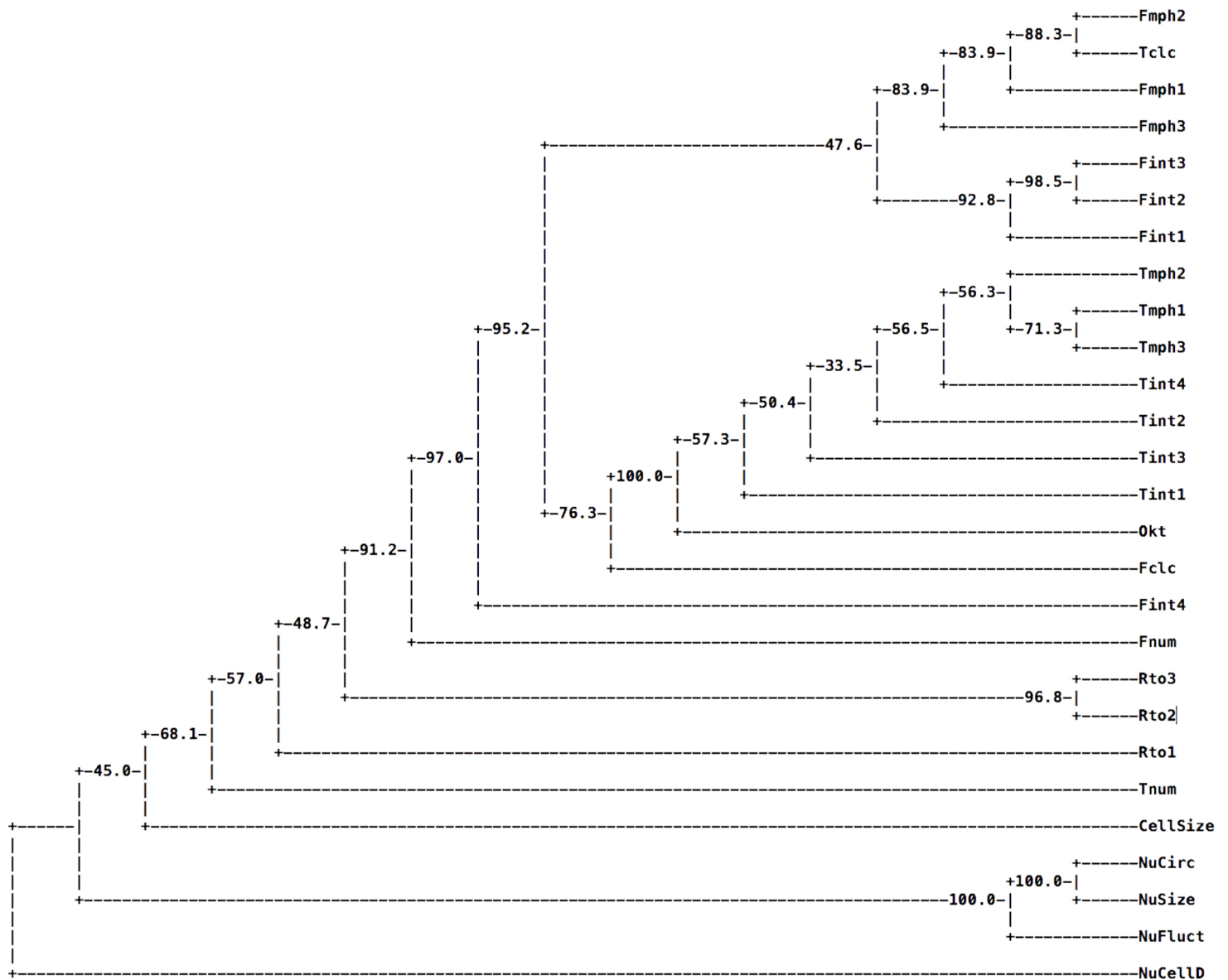

Supplement: Figure S5 — Bootstrap results for the primary tree. 100 replicate trees were generated by a half-jackknife operation, leaving out half the genes at random for each replicate. The final tree is a consensus of these 100 replicates. The bootstrap support for each branch (the fraction of replicates in which this branch occurs) is shown. (PDF) [file pone.0100554.s005.pdf]

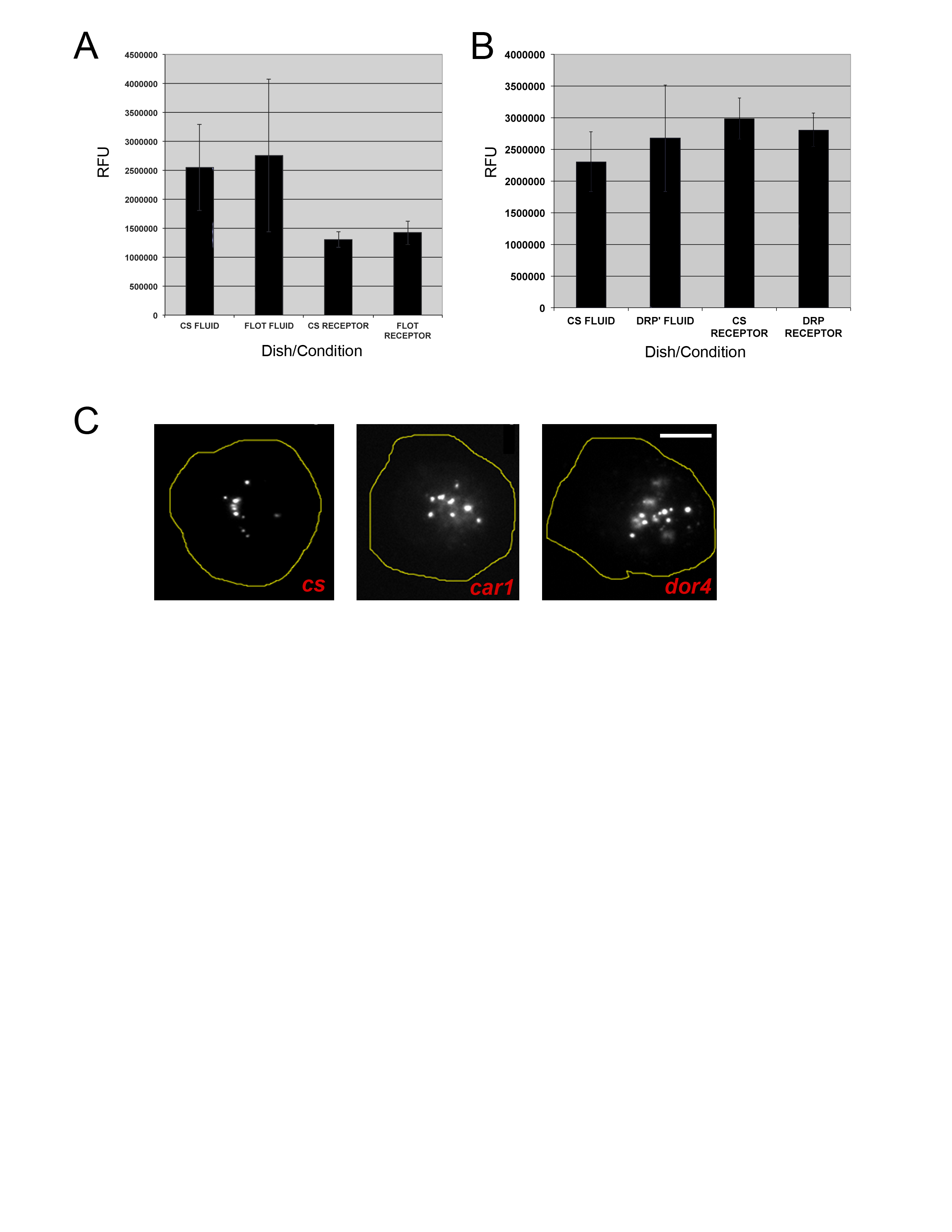

Supplement: Figure S7 — CG and CD pathways assessed in primary larval hemocytes from drp and Flo-1 mutants. (A,B) Fluorescent dextran (fluid) and anionic ligand binding receptor uptake of Cy3-malelylated BSA (receptor) in drp1 (DRP) and Flo-1 (FLOT) mutant hemocytes as compared to those from wild type CS flies. Graphs represent means ± SEM of relative fluorescence intensities (RFU) from 3 independent experiments (n = 10–20 cells per experiment). In each case, differences were not statistically significant from controls (Student T-test, p>0.05). (C) Representative micrographs of CS or mutant hemocytes (dor4, car1) that were pulsed for 3 min with fluorescent dextran and then chased for 12 min. As shown previously [47], these late endosomal mutants fail to traffic cargo to lysosomes and thus accumulate probe during the chase. See Figure 5B for quantifications. (TKF) [file pone.0100554.s007.tkf]

A

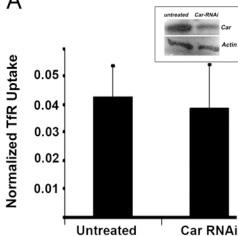

B

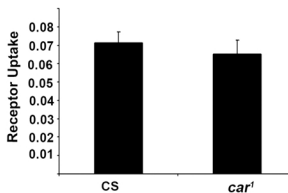

C

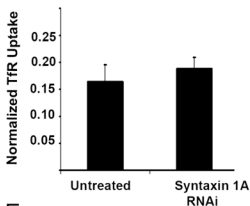

D

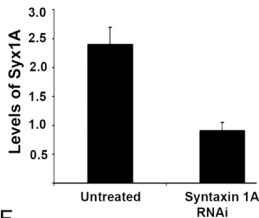

E

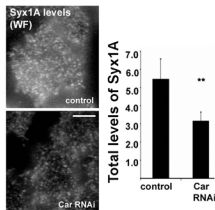

F

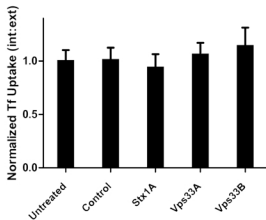

Supplement: Figure S8 — Effect of depletion of Car (hVps33) and Syntaxin 1 on levels of Syntaxin 1 and CD endocytosis. (A, B) Histograms show uptake of Tf (normalized to cell surface TfR (Okt9) staining levels) in Car-depleted S2R+ cells (A) and uptake of Cy3-malelylated BSA (receptor) in car1 mutant hemocytes (B). Note that there appears to be no significant difference in the uptake of bona fide CD-cargo in both cases when compared to control cells. Inset in A shows the protein levels of Car in the dsRNA-treated cells compared to untreated cells by western blot. Actin staining (at 40 kD) from the same western blots was used as a loading control. (C) Histogram shows that normalized Tf uptake in Syx1A-depleted S2R+ cells was no different from that measured in control cells as seen in a single representative experiment out of three independent experiments (n>50 cells per replicate, 2 replicates per experiment). (D) Levels of Syx1A were measurably different in Syx1-depleted cells (immunofluorescence, n>50 cells per replicate from 2 replicates). (E) The amount of Syx1A is reduced in Car-depleted cells. Graph represents normalized data from a representative experiment with n>50 cells per treatment from 2 replicates. (F) Histogram shows that normalized Tf uptake is unaffected in human AGS cells depleted of SYX1A, VPS33A and VPS33B. (PDF) [file pone.0100554.s008.pdf]
