# Supplementary material for: Population Distribution Analyses Reveal a Hierarchy of Molecular Players Underlying Parallel Endocytic Pathways
Source: PLoS One. 2014 Jun 27;9(6):e100554. doi: 10.1371/journal.pone.0100554 (PMC4074053; doi:10.1371/journal.pone.0100554)
Supplement: Figure S6 — Highlighted networks involved in protein degradation, the stress response, cell proliferation and the organization of the cytoskeleton. (A) Genes that affect both CG and CD pathways negatively (intersecting quadrants from Figure 5; see main text) were overlaid on a protein-protein interaction map (GeneMania; [84], with selected GO annotations highlighted in different colors (see legend). (B) Genes involved in cytoskeleton organization were selected using GO annotations, and were assayed for their roles in the CG pathway using the assay detailed in Figure 4. Complete scores are listed in Table S6. A network analysis [84] on the pooled set of results from this additional assay and the screen (Tables S1 and S4) highlights the significance of specific cytoskeletal elements in the two pathways of interest. The color of each edge in the network denotes the type of interaction (see legend below) and the fill color for each node denotes the functional category (see legend above network). Classification of the gene into a CG pathway hit, CD pathway hit, or a common hit is denoted by the letters F, T, or FT. (PDF) [file pone.0100554.s006.pdf]

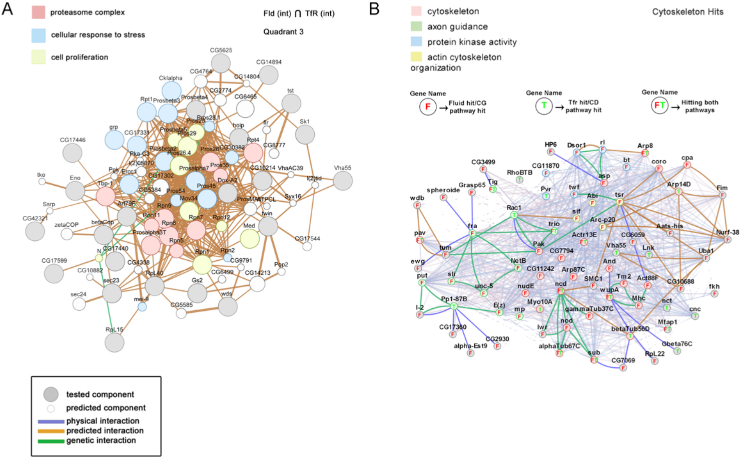
A
proteasome complex
cellular response to stress
cell proliferation
Fld (int) ∩ TfR (int)
Quadrant 3
CG5625
CG14894
CG4764
CG14804
CKIalpha
CG2774
tst
Rpt1
Prosbeta3
Prosbeta4
CG6465
Pros28.1
Pros25
grp
CG17331
Prosbeta5
Pros29
hoip
flr
Sk1
CG17446
Prosbeta2
Pros26.4
Pros26
CG30382
Rpt4
CG8777
Pka-C3
l(2)05070
Prosalpha7
Pros35
CG10214
VhaAC39
Vha55
tko
Eno
Pgk
Ercc1
CG17302
Dox-A2
l(2)tid
Tbp-1
CG5384
Pros54
Pros45
Mov34
ProsMA5
ATPCL
Syx16
Ssrp
Arl79F
Rpn9
Rpn11
Rpn7
Rpn12
twin
CG42321
zetaCOP
betaCop
Rpn6
Med
Prosalpha3T
Rpn5
CG17544
N
CG17440
Rpn1
Rpn2
CG17599
CG4338
CG9791
CG10882
RpL40
CG6499
CG14213
Pop2
sec23
Gs2
sec24
mei-9
wds
CG5585
RpL15
tested component
predicted component
physical interaction
predicted interaction
genetic interaction
B
cytoskeleton
axon guidance
protein kinase activity
actin cytoskeleton organization
Cytoskeleton Hits
Gene Name
F → Fluid hit/CG pathway hit
Gene Name
T → Tfr hit/CD pathway hit
Gene Name
FT → Hitting both pathways
HP6
Dsor1
rl
Arp8
CG3499
CG11870
bt
coro
cpa
RhoBTB
asp
Tig
Grasp65
Pvr
twf
Arp14D
spheroide
Abi
tsr
Fim
wdb
Rac1
sif
fra
trio
Arc-p20
Aats-his
pav
Nurf-38
Pak
Actr13E
CG6059
tum
Vha55
Lnk
Uba1
CG7794
NetB
And
ewg
CG11242
Arp87C
sli
Tm2
Act88F
put
SMC1
CG10688
unc-5
nudE
ncd
wupA
fkh
Pp1-87B
Mhc
nct
l-2
E(z)
Myo10A
mp
gammaTub37C
cnc
nod
Mfap1
CG17360
lwr
betaTub56D
CG2930
alpha-Est9
Gbeta76C
alphaTub67C
sub
RpL22
CG7069
