# Supplementary material for: Population Distribution Analyses Reveal a Hierarchy of Molecular Players Underlying Parallel Endocytic Pathways
Source: PLoS One. 2014 Jun 27;9(6):e100554. doi: 10.1371/journal.pone.0100554 (PMC4074053; doi:10.1371/journal.pone.0100554)
Supplement: Methods S1 — (DOCX) [file pone.0100554.s018.docx]

**­­­Supplementary Methods SM1**

**Endocytic assay, imaging and image analysis**

The assay for endocytosis (Figure **1A**) was adapted from existing protocols [[1](#_ENREF_1)]. Fdex and TMRdex, which are markers for bulk fluid uptake, were used at 1–2 mg/ml. Tf probes were used at 10 ug/ml. The salient feature of the assay is the simultaneous pulse-labeling of two endocytic pathways, using FITC-dextran for the CLIC/GEEC (CG) pathway (green in Figure **1C**) and A568-Tf (red in Figure **1C**) for the canonical clathrin-dependent (CD) endocytic pathway, followed by labeling of surface transferrin receptors with a labeled monoclonal antibody against the human transferrin receptor (A647-Okt9, blue in Figure **1B, 1C**). Surface labeling provides an estimate of transferrin expression levels and doubles up as a cell surface marker for image processing. The hemocyte receptor probe Cy3mBSA was used at 10–30 µg/ml in S2R+ cells. At these concentrations, the binding and internalization of labeled mBSA was completely competed by 100 fold excess unlabeled mBSA [[1](#_ENREF_1)]. Time points for the inhibitor treatments are as follows (these are all in CHO cells): For the test cases (drugs treated sequentially): 30 minutes of BrefeldinA (BFA) and 45 minutes of either Bafilomycin or Monensin, followed by 10 minutes of TMR-Dextran pulse.  Since these drugs are reversible they were maintained till the pulse was over.  Controls (BFA/Monensin/Bafilomycin single treatments) were treated to the equivalent time points as for the test situation.

The genome-wide screen was carried out on cell arrays (custom-designed glass slides) in a 300-well format (map in Figure **1A**). Each slide was seeded with 30 negative controls (15 with no dsRNA and 15 with scrambled DNA, black in Figure **1A**) and 8 positive controls (Sec23 and Arf1 for the GEEC pathway [[1](#_ENREF_1)] and Shibire (Shi) [[2](#_ENREF_2)] for the transferrin pathway, shades of blue in Figure **1A**), scattered across the slide. The rest of the wells contained dsRNAs targeting individual genes (white in Figure **1A**). Cells were allowed to grow on these slides for 4 days [[1](#_ENREF_1)] and then assayed as above. Each slide was assayed in triplicate, with the pattern scrambled for each replicate.

Sequential imaging of wells and image collection was carried out using two imaging modalities, a custom-built imaging station or a BD 800 pathway analyzer (Becton Dickenson). The custom built system includes a microscope stand (Nikon TE 300 and accessory optics; Nikon, Japan), precision controlled stage (Prior Scientific), and customized emission and excitation shutter wheels (Prior Scientific and Sutter Instruments) using device controllers (Ludl Instruments), and driven by customized autofocus-capable HCS software developed on open source microscopy control software, Micromanager^TM^. Images were acquired using a Nikon Plan Apo 0.75NA 20X lens. The results from the two imaging modalities were comparable in all aspects.

Single cells were identified using custom-written MATLAB routines that use the nuclear marker Hoechst and the surface marker Okt9 to determine cell outlines. Information was extracted from single cells in the form of various quantitative parameters (Supplementary Figure **S1B, C, D**). Intensity parameters describe total uptake levels of the two endocytic probes and steady state surface levels of the transferrin receptor (upper row of the table in Figure **1B**). Geometric parameters quantify the shape, size and number of endocytic compartments as well as the nucleus and the whole cell, extracted by thresholding the relevant intensity image and labeling individual compartments (lower row of Figure **1B**).

**A Z-score based on feature distribution shapes**

To quantitatively distinguish between distribution shapes, for the reasons outlined in the Main Text, we opted to use a modified version of the Kolmogorov-Smirnov (KS) test statistic for paired distributions [[3](#_ENREF_3)]. For each well and phenotypic parameter, we first affine-normalized the population distributions to have zero mean and unit variance. Given two such distributions, we calculated the maximum vertical distance between their cumulative distribution functions (the KS test statistic, *D*). As in the standard KS test, the statistic has to be scaled appropriately for differences in the number of cells and in the two wells being compared to one another. We defined an effective sample size . For effective sizes , we will have:


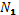

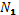

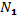

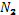

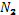

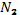

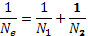

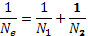

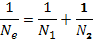

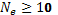

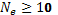

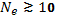


.


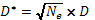

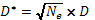

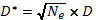


It should be noted that the statistic will not conform to the canonical KS distribution following the normalization procedure. Since we do not actually calculate the KS test *p*-value, this is not a critical issue.


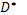

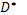


For each slide, we calculate the pair-wise for all 300 wells against the 30 negative control wells. To compensate for negative-to-negative differences, we normalize the values in each row of the resulting 30x300 matrix to have zero mean and unit variance. At this stage we set aside the 10 negative wells with the highest average values, to be used in later estimates of false-positive rates. The result is a 20x300 matrix, and the average value of each column is defined to be the Z-score of the corresponding well. Z-scores are low for negative controls and high for positive controls. When plotted in a heat map with a LUT bar ranging from black to white (low to high Z-scores), bright columns corresponding to test wells signal potential hits [[4](#_ENREF_4)]. Hits were selected by setting a threshold Z-score of 3 under which negative and positive controls are well separated.


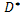

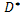

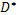

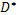

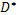

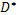


**References:**

1 Gupta GD, Swetha MG, Kumari S, Lakshminarayan R, Dey G, et al. (2009) Analysis of endocytic pathways in Drosophila cells reveals a conserved role for GBF1 in internalization via GEECs. PloS one 4: e6768.

2 Guha A, Sriram V, Krishnan KS and Mayor S (2003) Shibire mutations reveal distinct dynamin-independent and -dependent endocytic pathways in primary cultures of Drosophila hemocytes. J Cell Sci 116: 3373-3386.

3 Massey F (1951) The Kolmogorov-Smirnov Test for Goodness of Fit. Journal of the American Statistical Association 46: 68-78.

4 Dey G, Gupta GD, Ramalingam B, Sathe M, Mayor S, et al. (2014) Exploiting cell-to-cell variability to detect cellular perturbations. PloS one 9: e90540.
